# Materials Research Directions Toward a Green Hydrogen Economy: A Review

**DOI:** 10.1021/acsomega.2c03996

**Published:** 2022-09-09

**Authors:** Zachary J. Baum, Leilani Lotti Diaz, Tatyana Konovalova, Qiongqiong Angela Zhou

**Affiliations:** CAS, a division of the American Chemical Society, Columbus, Ohio 43202, United States

## Abstract

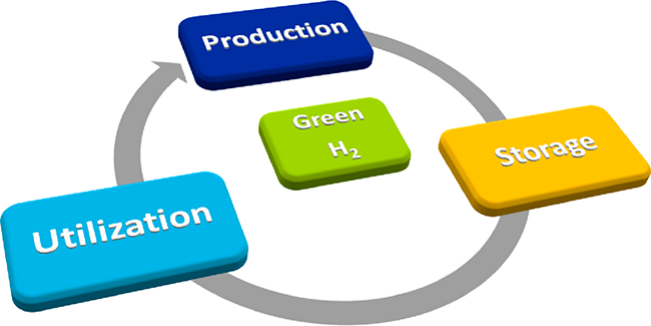

A constellation of technologies has been researched with an eye toward enabling a hydrogen economy. Within the research fields of hydrogen production, storage, and utilization in fuel cells, various classes of materials have been developed that target higher efficiencies and utility. This Review examines recent progress in these research fields from the years 2011–2021, exploring the most commonly occurring concepts and the materials directions important to each field. Particular attention has been given to catalyst materials that enable the green production of hydrogen from water, chemical and physical storage systems, and materials used in technical capacities within fuel cells. The quantification of publication and materials trends provides a picture of the current state of development within each node of the hydrogen economy.

## Introduction

Despite decades of research toward alternatives, fossil fuels account for more than 80% of the global energy consumption today.^1^ Facing dwindling natural resources and burgeoning ecological consequences, we are challenged with charting a sustainable course for modern life using renewable energy sources. This will require safe and reliable methods of converting, storing, and using energy that can compete with hydrocarbon fuels extracted from the Earth. While the optimal solutions may vary depending on the geographical location and availability of alternative energy-enabling materials, one major proposed avenue is the use of hydrogen as an energy carrier and hydrogen fuel cells as a primary method of converting energy into electricity. The integrated system of hydrogen production, storage, and utilization on a societal scale is aspirationally referred to as the *hydrogen economy*.

Hydrogen has the potential to act as a superior energy carrier when compared to fossil fuels, as it has approximately twice the gravimetric energy density and could have none of the carbon emissions.^2^ However, the primary mode of producing hydrogen today is from the reforming of fossil fuels (natural gas, oil, and coal), which together account for 96% of production.^3^ This is hardly a solution for enabling sustainable energy. However, there is a cleaner and “green” alternative to produce hydrogen, by using water electrolysis with the help of renewable energy sources. In this Review, we refer to the integration of renewable hydrogen into the global energy system as the *green hydrogen economy* (GHE) and explore research trends in each of the three facets of the green hydrogen economy: green hydrogen production, hydrogen storage, and hydrogen-based fuel cells. Using data from the CAS Content Collection, we analyze the academic and patent literature from 2011 to 2021 to understand the general progress of each field as well as the classes of materials and concepts driving their innovation (see search method and data in the Supporting Information). As an expert-curated resource, the CAS content is utilized here for the quantitative analysis of publications against variables including time, country/region, research area, and substance details. We hope that this Review serves as a broad overview of the materials research directions driving the potentially transformative set of GHE technologies.

## Hydrogen Production

### Production by Water Electrolysis

Efforts toward renewable hydrogen production center on water electrolysis, where water is split into hydrogen and oxygen using electricity. In general, water electrolyzers consist of two electrodes, an anode and a cathode, dipped in water and separated by a semipermeable separator.^4^ An external electrical circuit connects the electrodes to a power source. Water enters the electrolyzer and is subjected to electrical current, causing it to split into hydrogen and oxygen. A reduction occurs at the cathode to produce H_2_, and an oxidation occurs at the anode to produce O_2_. These two reactions are respectively referred to as the oxygen evolution reaction (OER) and the hydrogen evolution reaction (HER) and proceed according to the following equations in acidic media:
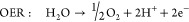
1
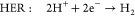
2
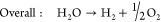
3

**Figure 1:**
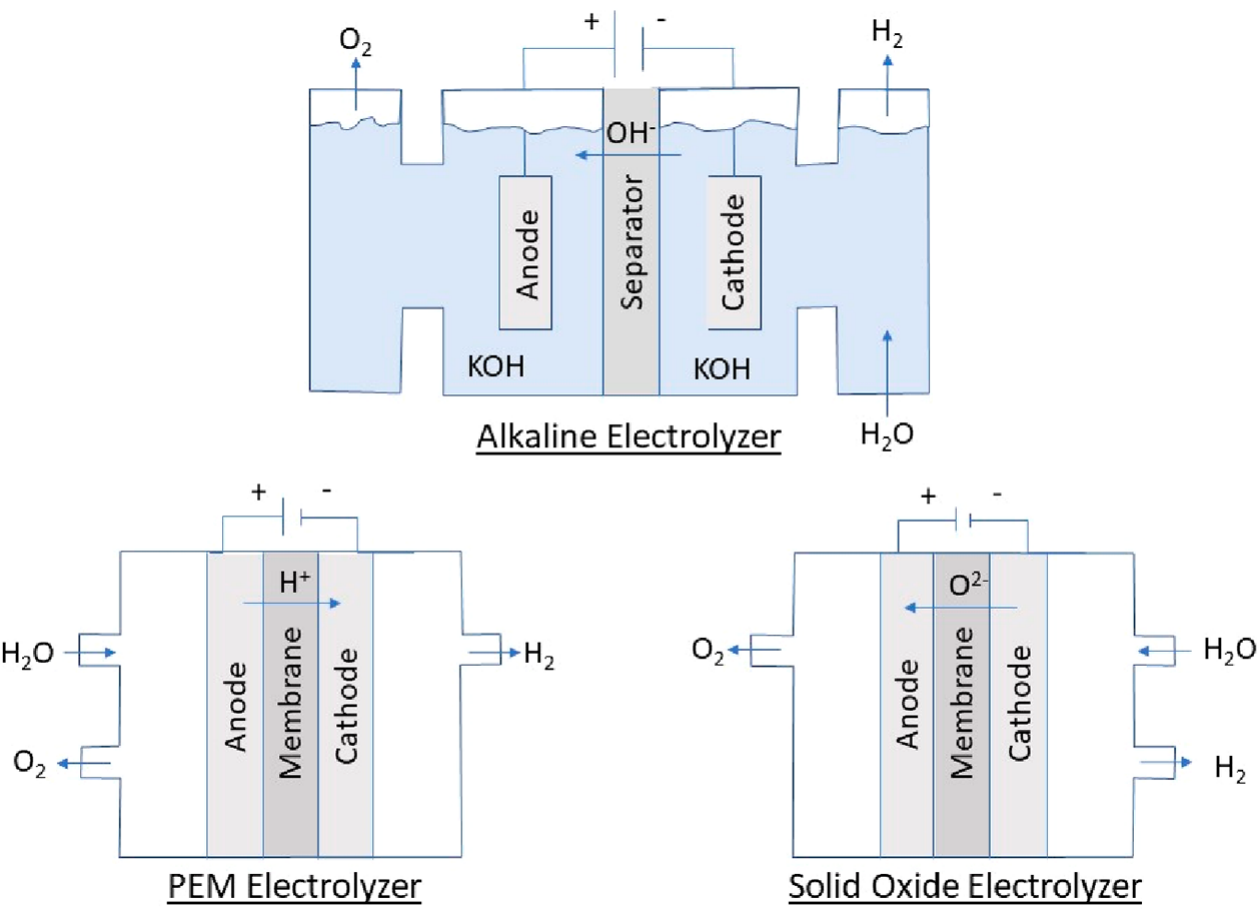
Electrolyzer configurations of interest for application.

Electrocatalysts, usually platinum group metals, are needed to reduce the overpotential of the electrochemical reactions by adsorbing reactants on their surface to form intermediates that promote the charge transfer in the electrolyzer.^5^ These chemical principles can be applied in various electrolyzer configurations to produce H_2_ from water. The three primary technologies of interest for industrial applications are alkaline electrolyzers (AEs), proton exchange membrane electrolyzers (PEMEs), and solid oxide electrolyzers (SOEs), all shown schematically below in Figure 1.

Alkaline electrolyzers contain either KOH, NaOH, or NaCl as a base and an electrolyte in a tank of water and have an anion-permeable separator (for example, asbestos or Zirfon) or an anionic polymer membrane.^4−6^ The reactions that take place at each electrode are the following:
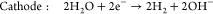
4
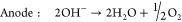
5
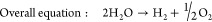
6Water enters the cathode where it is split into H_2_ and OH^–^ anions (Figure 1). The separator allows only the OH^–^ anions to transfer from the cathode to the anode to be oxidized into oxygen.^4^ Water is continuously added to the tank as hydrogen and oxygen are recovered from the splitting reaction.

AEs have many advantages when compared to PEMEs because of their alkaline nature. They have less corrosion problems than acidic electrolyzers, allowing the use of less expensive and more abundant catalysts than Pt (for example, Ni, Co, Fe, Mo, or Zn), longer lifetimes, and lower maintenance costs.^3,7^ Though AEs are a mature and globally commercialized technology, they still have disadvantages: HER is more sluggish in an alkaline medium, they have limited current densities, low operating pressures, and low energy efficiency (70–80%), and they must be adapted to a more dynamic operation to work with energy-fluctuating renewable sources.^5−7^ Santos et al. have recently presented options for the optimization of AEs that address some of the materials challenges in this technology: in particular, the porosity and wettability of electrodes to facilitate the detachment and surface coverage of gas bubbles, enhancement of the ion conductivity of the electrolyte via additives, and alternative diaphragm or separator materials.^3,4^

Polymer exchange membrane electrolyzers are acidic in nature and contain a polymer electrolyte membrane that is permeable to protons. The reactions that take place in the electrodes are the same as eqs 1–3, with water entering the anode where the OER takes place. The hydrogen cations pass through the membrane to the cathode where the HER takes place to produce H_2_ (Figure 1). Perfluorosulfonic acid polymer membranes such as Nafion, Fumapem, Flemion, and Aciplex are typically used.^6^

PEMEs are more compact than AEs, have high current densities and efficiencies (80–90%), produce hydrogen and oxygen of higher purity, and have high dynamic operation, making them more compatible with renewable energy sources.^6^ However, the acidic environment of PEMEs requires the use of expensive noble metal catalysts (Pt/Pd for HER and IrO_2_/RuO_2_ for OER), while their susceptibilities to membrane contamination and anode deterioration cause durability and lifetime issues.^3,6^ The development of nonprecious metal catalysts that meet the activity and stability requirements, catalyst supports to reduce loading, and the development of anticorrosion bipolar plates are the materials challenges being researched.^8^

Solid oxide electrolyzers are unique as they operate at high temperatures (500–1000 °C) to allow the dense ceramic electrolyte layer, usually yttria-stabilized zirconia, to conduct oxygen anions or hydrogen protons.^9^ The typical reactions that take place at each porous electrode are the following:
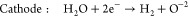
7
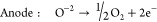
8
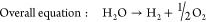
9Water enters the cathode, usually a composite of Ni and YSZ or a perovskite-like material, where it is split to produce H_2_ and oxygen anions that move through the solid electrolyte to the anode (perovskite-based like lanthanum strontium cobalt ferrite) to produce molecular oxygen. Proton-conducting SOEs operate with the same working principle as PEMEs (eqs 1–3).^10^

SOEs, with their high temperatures, have the advantages of more favorable thermodynamics and faster kinetics than AEs and PEMEs.^11^ They need less electrical energy to split water, operate at high current densities, produce pure hydrogen, and have higher efficiency.^9,12^ Though this technology is promising, it faces big challenges when it comes to the durability of the materials due to the high operating temperatures.^9^ Nechache et al. and Hauch et al. discuss alternative cell materials and recent advances in more detail, for example: lanthanum strontium gallium magnesium (LSGM) as the electrolyte, emerging the metal catalyst from the backbone and to the surface of the perovskite electrolyte by applying voltage (metal-exsoluted perovskite) for the cathode, and nickel-based materials for the anode.^11,12^

As briefly discussed, materials challenges motivate research in each of these electrolyzer categories. For example, minimizing internal resistances, optimization of the membrane–electrode assembly, and selection of separator material all play into the device efficiency.^3^ With these in mind, research on the catalyst components of electrolyzers is more active and will be considered in this Review from a critical materials perspective.

Just as with energy stored in battery electric vehicles, water electrolysis-produced hydrogen is only as green as the electricity used in its generation. Lifetime assessment studies done by Bhandari et al. revealed that electrolytic hydrogen production using renewable energy-based electricity (wind, solar, hydropower, or biomass) can reduce total carbon emissions by more than 90%.^13^ Considering the amount of generated electricity and the materials required to build electrolyzers, wind turbines, or solar panels, wind energy-based electrolysis is proved to be the best technology for H_2_ production.

### New Directions in Hydrogen Production

**Figure 2:**
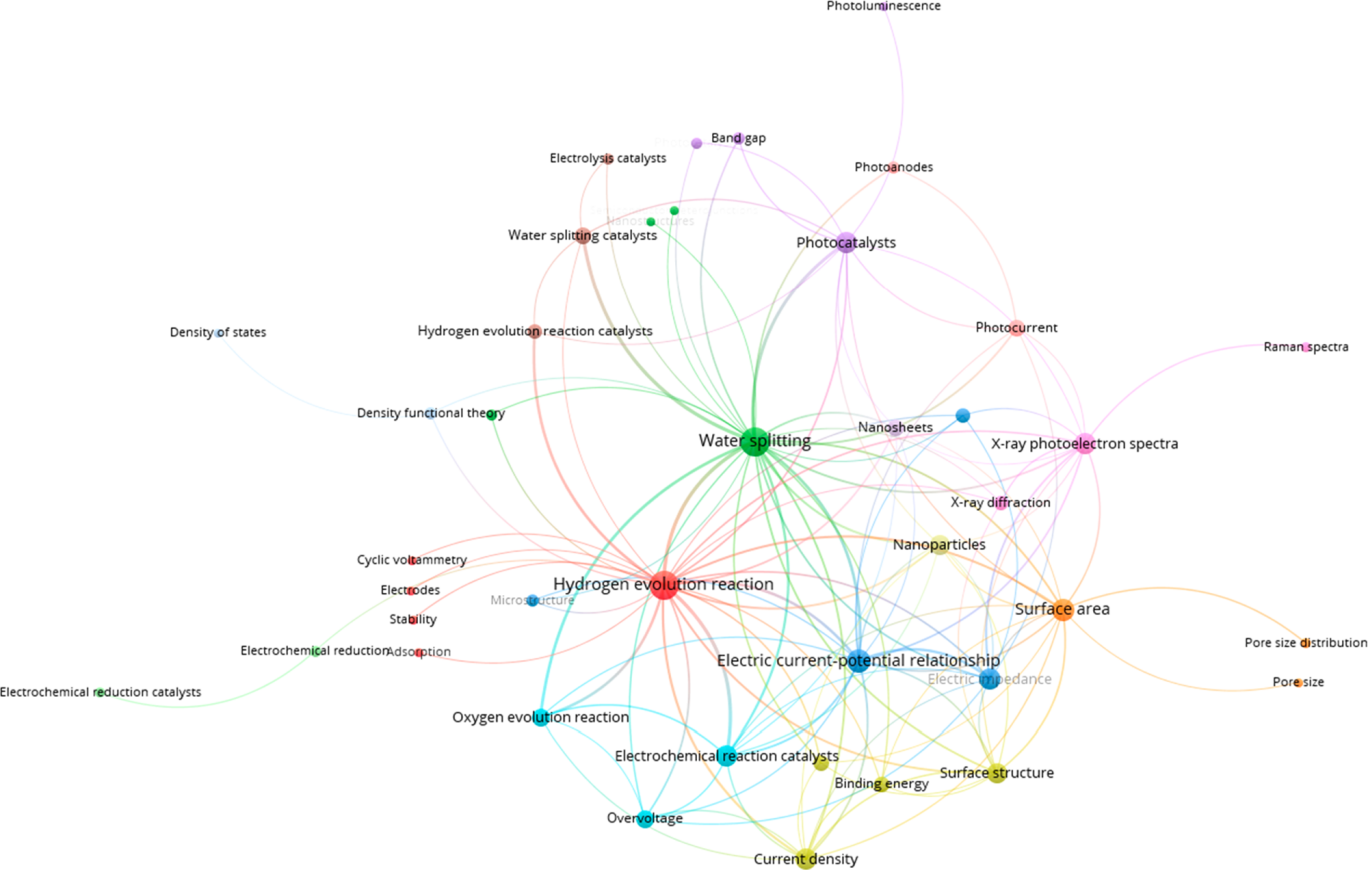
Top 125 pairs of co-occurring concepts in the green hydrogen production literature from 2011 to 2021.

Water electrolysis is an energy-intensive process that benefits from the use of catalysts. Because the canonical hydrogen evolution catalyst is Pt and the oxygen evolution catalyst is RuO_2_, an important research focus for green hydrogen production has been the development of catalysts that rival scarce metals in performance but with reduced or eliminated metal loading.^14,15^ The recent materials research landscape in this area can be visualized in many ways; we begin by presenting the most commonly co-occurring concepts found to be important within each respective study in a clustered network diagram generated by VOSviewer (Figure 2).

From this high-level conceptual analysis, we see that “hydrogen evolution reaction” and “water-splitting” concepts are indexed with similar frequencies, while “oxygen evolution reaction” commonly co-occurs as well. This finding underscores that studies of overall water splitting must consider both OER and HER to reach a complete understanding.^16,17^ In addition, “photocatalysts” are a common research theme from 2011 to 2021 and have significant overlap with several nanomaterial-related concepts; the fact that photocatalysts co-occur at approximately the same rate as electrochemical reaction catalysts demonstrates how important photocatalysts have become in the field. Finally, the inclusion of a cluster of surface-oriented concepts such as “surface structure”, “surface area”, and “pore size” shows the relevance of surface phenomena in catalyst design.

**Figure 3:**
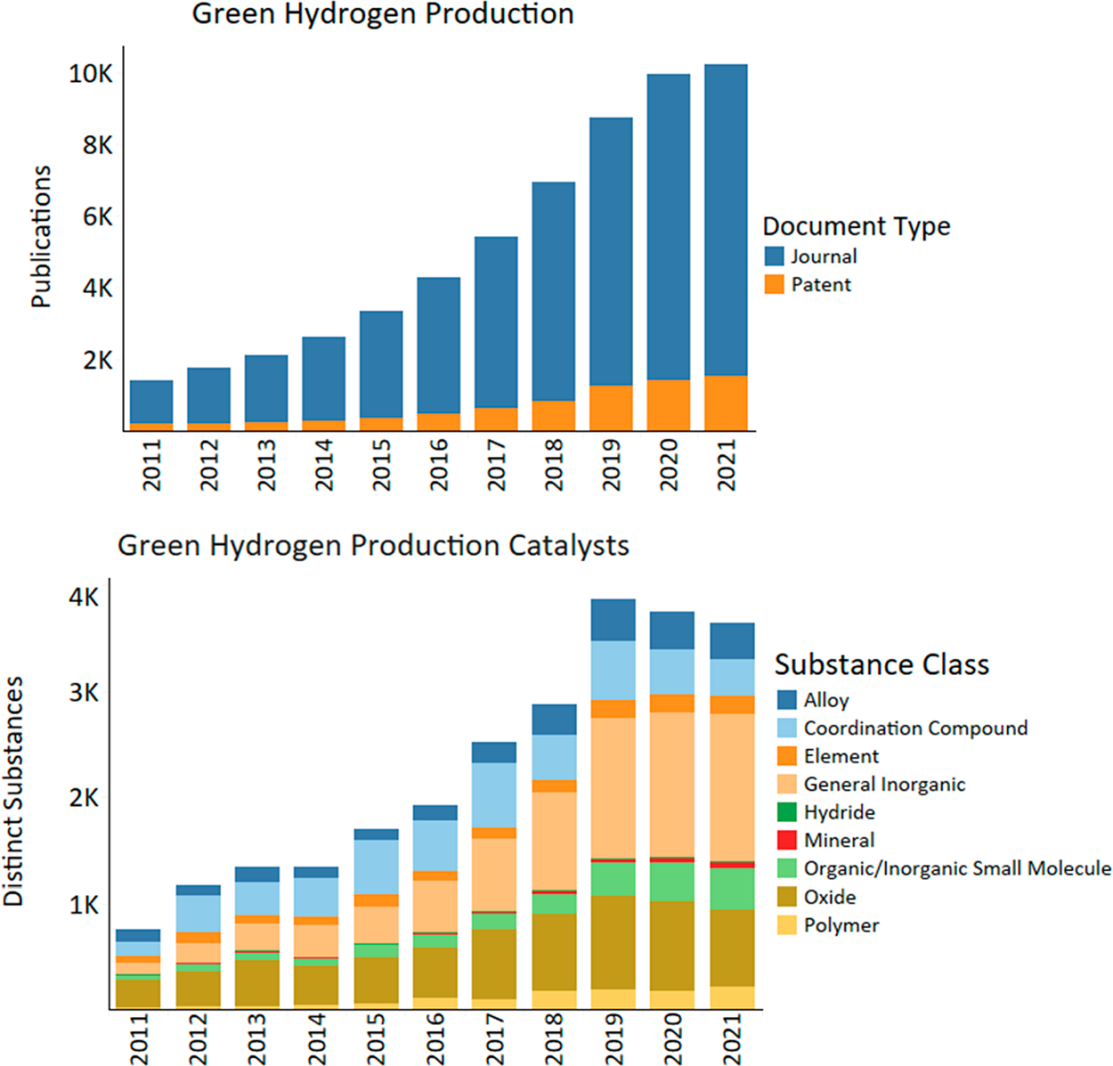
Publication trends and distinct substances used for catalysts by year in green hydrogen production research from 2011 to 2021.

Between 2011 and 2021, an over fivefold increase of publication volume was observed in green hydrogen production (Figure 3). This is driven by a concomitant increase in both journal articles and patents. After experiencing rapid growth throughout the decade, the publication volume appears to be leveling off.

Green hydrogen production was discussed mainly in journals, but the fraction of patent documents increased over the second half of the decade, reaching 15% of the total publication volume in 2021.

Concerning catalytic materials in green hydrogen production journal articles and patents, the scope of several substance classes expanded appreciably in the 2010s and culminated in a peak of substance diversity in 2019 (Figure 3). The slight dip in distinct substances from 2019 to 2021 contrasts with the continued overall increase in publication count. On the basis of these observations, green hydrogen production catalysis appears to be reaching maturity as a research field with commercial potential.

**Figure 4:**
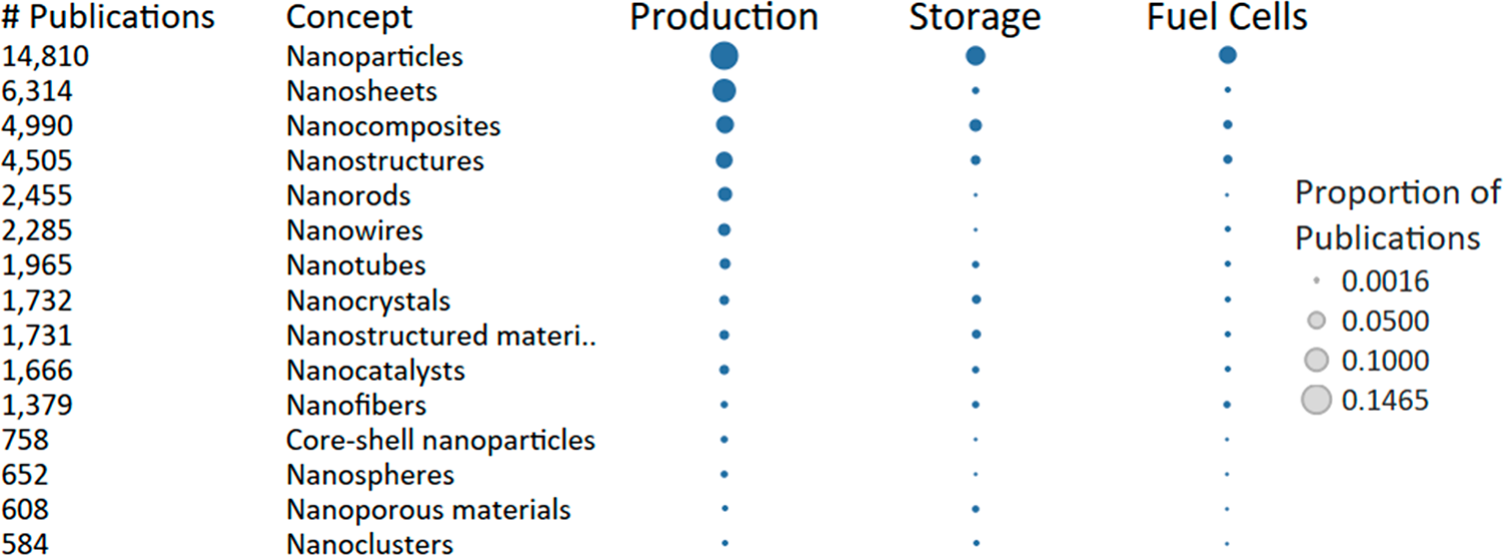
Top nanotechnology-related concepts in each area of GHE research from 2011 to 2021.

The relative prevalence of the most common nanomaterial types in GHE research are shown in Figure 4, normalized to the number of publications in each respective research area. For green hydrogen production, the “nanoparticles” concept is the most common, followed by “nanosheets” and “nanocomposites”. The popularity of nanoparticles is well-known, with Pt nanoparticles being considered among the top-performing HER electrocatalysts. Concerning the almost equally popular nanosheets, materials chemistry has been enamored with these two-dimensional materials for the last 15 years, and a diverse set of products can be prompted to form into atomically thin dimensions to give rise to novel and useful phenomena for catalysis.^18^ When combined with other materials (into nanocomposites), high surface area catalysts can be prepared which take advantage of nanoscale effects such as quantum confinement^19^ and surface plasmon resonance,^20^ as well as interfacial effects including the aforementioned semiconductor heterojunctions and Schottky junctions.^21^ In addition to the transition metal dichalcogenides^22^ (e.g., MoS_2_) and graphitic carbon nitride^23^ (C_3_N_4_) mentioned in Table 3, nanosheets used for green hydrogen production have thus far included layered double hydroxides,^24^ graphene,^25^ MXenes,^26^ bismuth oxyhalides,^27^ halide perovskites,^28^ and 2D MOFs^29^ and covalent–organic frameworks.^30^ The vast selection of electronic materials available and the toolbox of synthetic methods impart control over particle size, shape, doping and defect levels, crystallinity, and material interfaces, thus motivating the large number of observed studies on nanoscale morphology in green hydrogen production.

The substance information found within publications provides additional insights. While publication volume alone provides a picture of relative research interest over time, it does not detail the diversity of approaches being taken on a given topic. Analysis of relevant substance classes within this progression over time reveals several research trends (Figure 3). For example, the increase in compounds of the classes alloy and element shows the exploration of chemical space for alternative electrocatalysts to Pt^31^ and as components in composite materials as electro- and/or photocatalysts. Coordination compounds, on the other hand, saw an increase in material diversity in the first half of the decade followed by sustained research interest. During this period of time, metal–organic framework (MOF)-based and MOF-derived materials saw increased interest in heterogeneous catalysis.^32^ The application of semiconductor engineering to photocatalysis saw compounds of the classes general inorganic and oxide increasingly applied to green hydrogen production throughout the decade alongside their use as catalyst supports.^33,34^ Finally, polymers began to be studied as components in heterojunction catalysts,^35^ as tunable stand-alone porous photocatalysts (in the case of covalent organic frameworks),^36^ and as precursors to engineered carbonaceous catalyst materials.^37,38^ The top-studied materials from these classes in 2021 are shown below in Table 1 alongside their respective important research focus.

**Table 1:** Key Substances in Green Hydrogen Production Catalyst Research

catalyst substance class	substance	REG #	2021 publications	feature(s)	exemplary publications
oxides	RuO_2_	9002-89-5	185	standard for comparison for OER but also frequently used in nanocomposite electrocatalysts	(40−42)
	TiO_2_	13463-67-7	421	catalyst support; frequently doped and/or heterostructured nanocomposites for both photocatalysis and electrocatalysis	(43−45)
general inorganics	C_3_N_4_	143334-20-7	477	facile synthesis into nanostructures, amenable to vacancy engineering for photocatalysis	(46−48)
	MoS_2_	1317-33-5	308	exfoliatable semiconductor nanosheets for photocatalysis	(49−51)
elements	carbon	7440-44-0	917	prepared via various sources to control morphology and doping level of a (photo)electrocatalyst component	(52−54)
	platinum	7440-06-4	899	nanostructured or “single-atom” catalysts for decreased Pt loading in HER	(53, 55, and 56)
	nickle	7440-02-0	681	Ni foam as an electrocatalyst component; in situ transformations into active nanocatalyst components; single-atom catalyst studies	(54, 57, and 58)
coordination compounds	UiO-66(NH_2_)	1260119-00-3	11	visible light-responsive porous photocatalyst component	(59−61)
	ZIF-67	46201-07-4	27	doped, surface-engineered, and/or calcined to produce novel Co-based (photo)electrocatalysts	(62−64)
alloys	iron–nickel alloy	11148-32-6	64	electrodepositable nanocomponent in overall water-splitting electrocatalysts	(65−67)
	cobalt nickel alloy	11101-13-6	43	nanocomposite electrocatalysts with other top materials	(68−70)
polymers	polyaniline	25233-30-1	15	conductive polymers in nanocomposite (photo)electrocatalysts	(71−73)
	polypyrrole	30604-81-0	13		(72, 74, and 75)

As detailed in Table 1, the control and exploitation of nanoscale morphology are currently areas of heavy focus for green hydrogen production catalysts. Several factors play into this research focus. First, it is desirable in electrocatalysis to maximize the electrochemically active surface area of the catalyst to increase the number of active sites available and, in photocatalysis, to maximize light absorption. In addition, the dispersal of precious metals onto a nanostructured surface can improve the catalytic properties on an atomic metal basis. Finally, in heterostructured semiconductor photocatalysts as well as Schottky junction materials, the management of variables such as exciton separation, carrier diffusion, and mass transfer becomes quite complicated.^39^

**Figure 5:**
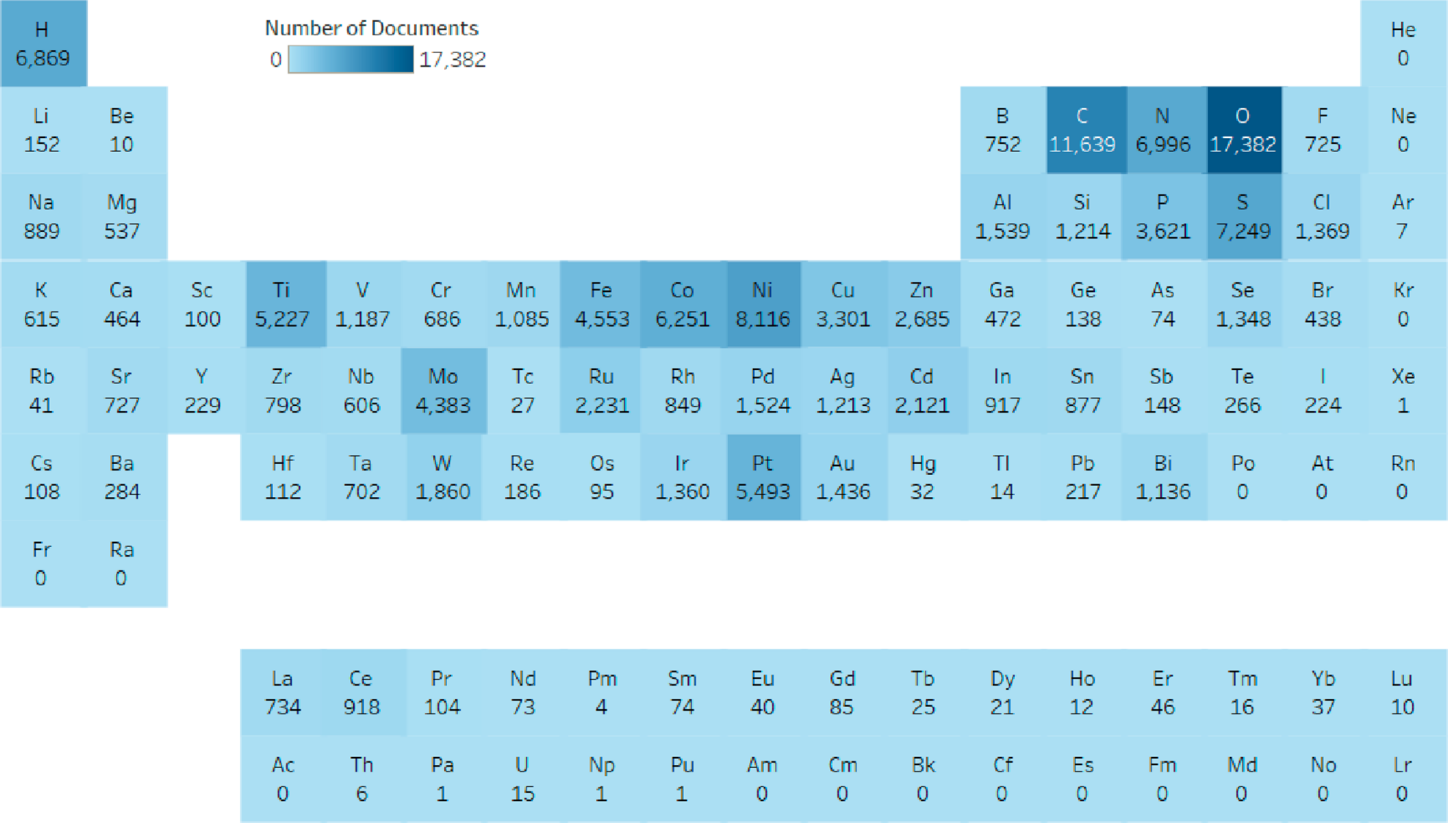
Occurrence of elements in materials used as green hydrogen production catalysts by number of documents from 2011 to 2021.

We also consider here the relative prevalence of elements in catalysts used for green hydrogen production on a document-level basis, shown below in Figure 5. This provides a rough landscape for assessing the overall elemental distribution of research interest and may point to future resource requirements for adoption on a societal scale. Overall, emphases on carbonaceous materials as well as transition metal oxides and sulfides are evident. There has been strong interest in critical metals including cobalt, nickel, and platinum, with the peak publication volume centered at the expected d^8^ transition metals typical of HER catalysts.

### Hydrogen Storage

Hydrogen is a desirable energy storage carrier, as it has the highest energy per mass (142 kJ/g) of any fuel.^76^ The development of the GHE has been hindered by the difficulty in storing hydrogen. Its low ambient temperature density results in a low energy per unit volume, therefore requiring the development of advanced, safe, and efficient storage methods with the potential for high energy density.^77^

### Physical-Based Storage

Hydrogen can be stored in several ways including physical-based and chemical-based storage. Conventional hydrogen storage methods include compression, liquefaction, and cryo-compression.

#### Compressed Hydrogen

The compression of hydrogen into tanks provides the benefits of quick charge and discharge times. However, the volumetric density of hydrogen is much lower than that of other energy sources, for example, four times lower than that of natural gas. Therefore, hydrogen needs to be compressed to extremely high pressures (700–800 bar or higher) to achieve reasonable volumetric density.^78^ Thick-walled tanks made of carbon fiber composites with steel or aluminum (Type-3) or polymer linings (Type-4) therefore become necessary. The tanks are accompanied by inherent safety risks including explosion and fire and are troubled by the permeability of hydrogen and the embrittlement of the tank walls. A recent study of 350 and 700 bar polymer-lined H_2_ storage tanks has shown that carbon fiber-reinforced composites are needed to provide the structural strength for these fuel tanks. For 700 bar H_2_ storage tanks, high-density polyethylene (HDPE) liners fully wrapped with carbon fiber composites are required.^79,80^

#### Liquified Hydrogen

Liquefaction is an option if an application requires the hydrogen volume to be reduced further than compression can achieve.^78^ The Mitsubishi Heavy Industries Group and the space industry have used liquefied hydrogen to fuel rockets for many years. However, liquid hydrogen storage is energy intensive, technically complex, and very costly. Hydrogen must be cooled to −253 °C and stored in insulated tanks to maintain this extremely low temperature. To minimize the losses due to vaporization, transfer of liquid hydrogen should be performed in a vacuum-insulated system. In addition, this transfer should be conducted in a closed system with a proper safety relief device to avoid a flammable atmosphere or an explosive mixture of air and liquid hydrogen.^81^

#### Cryo-compressed Hydrogen

The two techniques—compression and liquefaction—can also be combined. A volumetric density of 70.8 kg/m^3^ can be achieved using 54.7 kJ/g of energy as work through a process of compression and cooling in cryogenic tanks while the gravimetric density is influenced by the tank size. In Type 3 (metal-lined) cryogenic tanks, a 2 mm stainless steel liner meets the 15 000-fatigue cycle life requirement for storage pressures up to 700 bar.^82^

A recent study by the UK government found that, though hydrogen is not a direct greenhouse gas, it has a global warming potential of 11 ± 5 owing to changes in the concentrations of the important greenhouse gases in the atmosphere.^83^ H_2_ leakage of physical-based storage is therefore a great environmental concern. Physical storage of H_2_ also does not typically meet applicable safety and/or density requirements, especially for transport applications; therefore, chemical hydrogen storage methods have received widespread attention.

### Material-Based Storage

**Figure 6:**
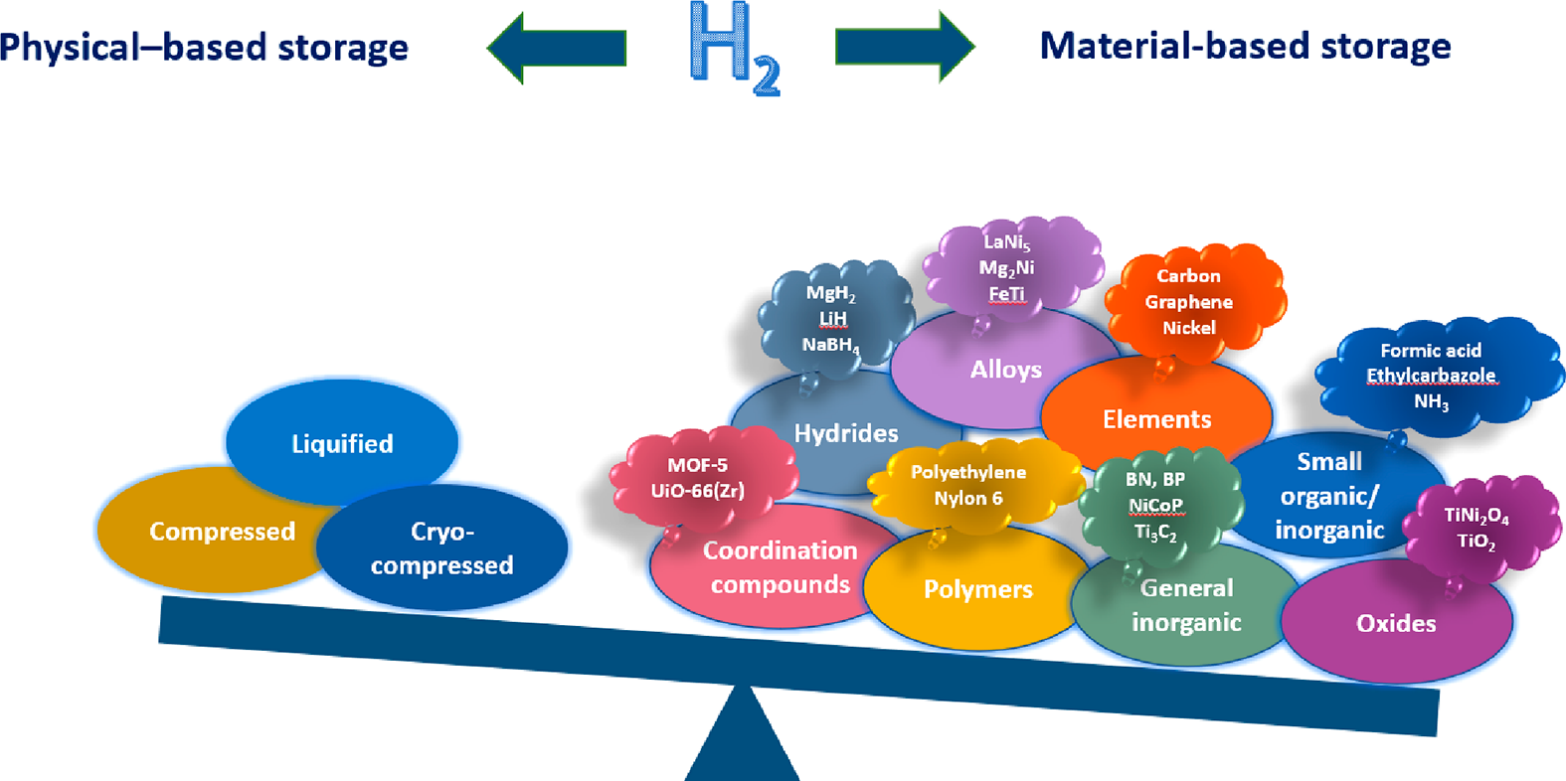
Research activity: physical vs chemical hydrogen storage.

Hydrogen storage materials can be divided into two categories based on the relative strength of the material interaction with hydrogen: physisorption materials and chemisorption materials (Figure 6). In physisorption materials, H_2_ molecules are adsorbed via a weak van der Waals interaction on the surface of the pores. The physisorption process is reversible because the interaction energy is low. The dominant materials in this class are carbonaceous sorbents where physisorption is proportional to their specific surface area.^84,85^ Storage pressures for physisorption-based sorbent systems can be much lower than for physical storage methods without a significant reduction in capacity. Storage temperatures can also be higher, thus reducing the cost for insulation and the energy consumption for cooling. Lastly, it is a completely reversible process and does not require off-board regeneration as is needed for chemical storage options. The main challenge for physisorption materials is the low binding energy for H_2_. An approach is to use cryogenic temperatures to enhance gravimetric and volumetric storage capacities of physisorbents, although this comes at the cost of some of the benefits.^79^

In chemisorption-based materials, hydrogen chemically interacts with the storage medium. The chemisorption process may not be fully reversible owing to the high activation energy in the adsorption and desorption process. On-board hydride materials belong to this class.^86,87^

**Figure 7:**
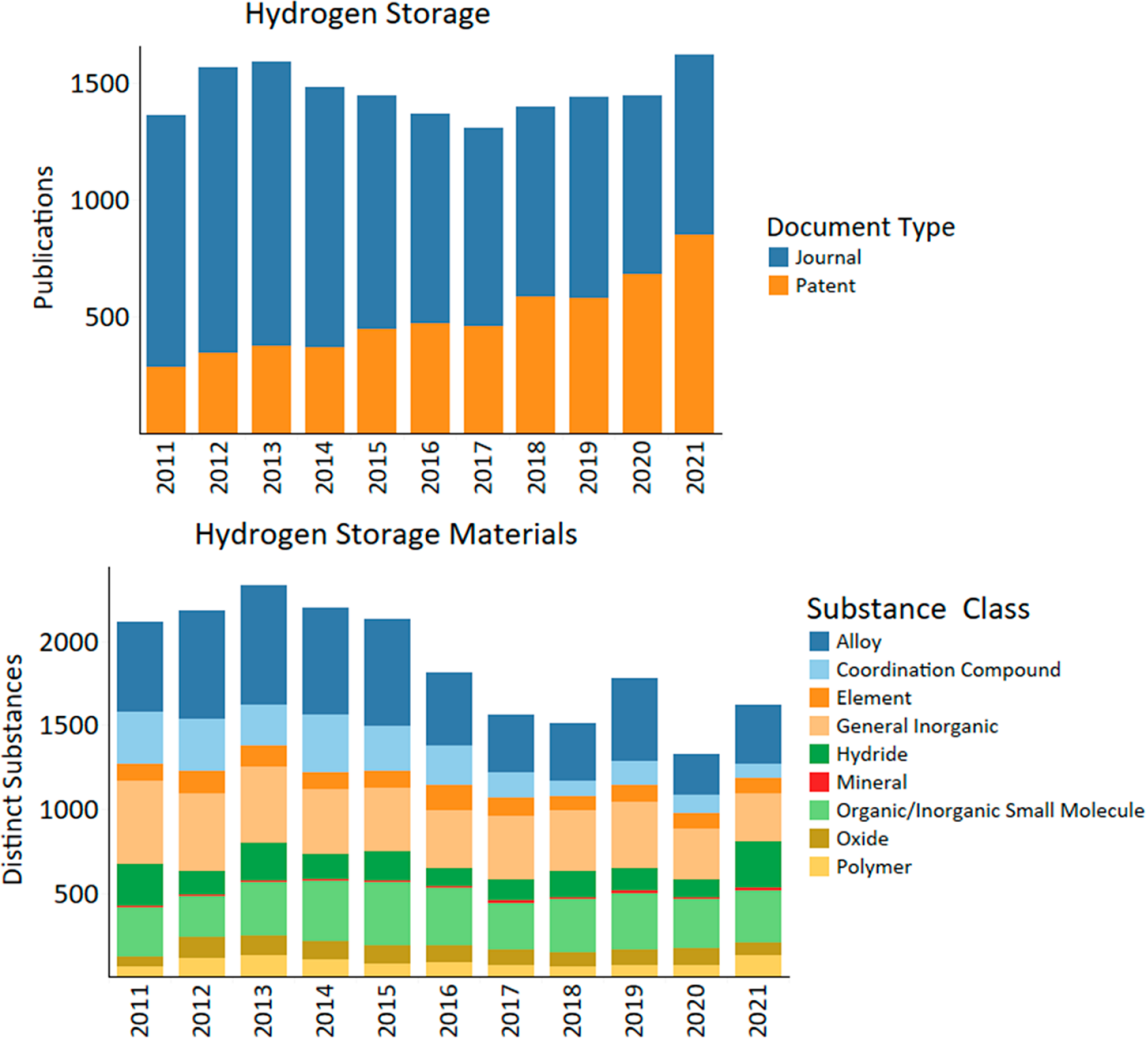
Publication trends and distinct substances used for material by year in hydrogen storage research from 2011 to 2021.

In contrast to green hydrogen production, the number of annual publications on H_2_ storage is steady at about 1500 with yearly fluctuations between 2011 and 2021 (Figure 7). However, this total hides the declining number of journal publications. The surge of the 2012–2013 publications coincides in time with the first commercially produced hydrogen fuel cell vehicle, Hyundai ix35 FCEV, introduced by Hyundai in 2013.^88^ A decrease in journal publications occurred up to 2017. At the same time, the number of patents shows a steadier growth with fewer fluctuations. The growth of patents may indicate a special interest of manufacturers such as Toyota, Honda, Hyundai, and Panasonic in developing new on-board H_2_ storage technologies (Table 5).

**Figure 8:**
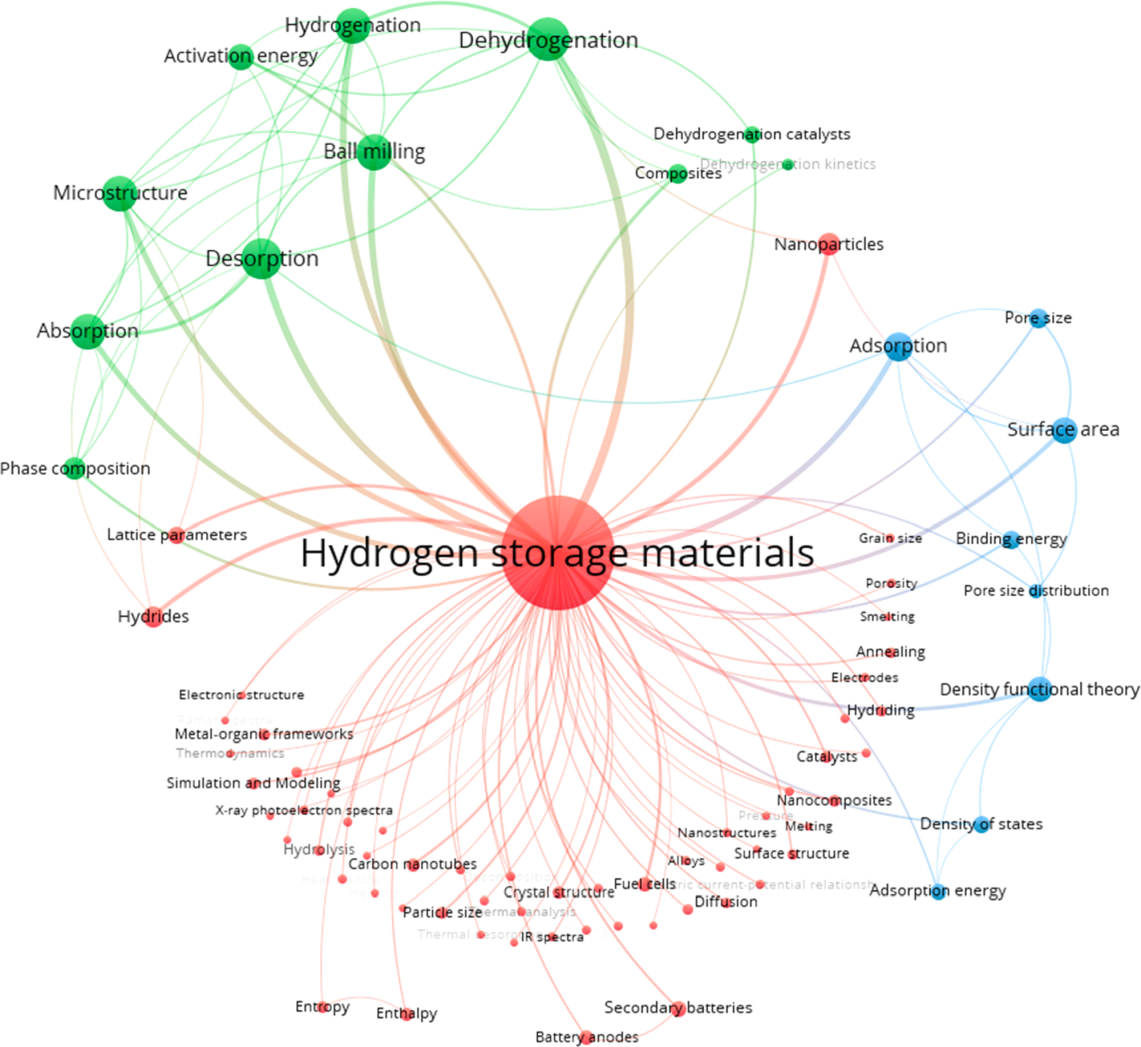
Top 125 pairs of co-occurring concepts in the hydrogen storage literature from 2011 to 2021.

The progress in the H_2_ storage research is illustrated by the concept cluster map shown in Figure 8. “Hydrogen storage materials” are the key concept of the map. The concept is commonly used together with “dehydrogenation,” “hydrogenation,” “dehydrogenation catalysts,” and “hydrides”, indicating H_2_ storage and release via chemisorption. “Hydrogen storage materials” frequently co-occur with “absorption,” “desorption,” “metal–organic frameworks,” and “carbon nanotubes”, which indicate physisorption and common physisorbents. “Microstructure,” “ball milling,” “nanoparticles,” and “nanostructures” demonstrate current trends in material modification; they often co-occur with “activation energy,” “surface area,” “pore size,” “pore size distribution,” and “binding energy.” Finally, “fuel cells” and “secondary batteries” indicate possible commercial uses for H_2_ storage.

The most frequently reported substance classes for hydrogen storage selected from 2011 to 2021 publications are summarized in Figure 7. The development of hydrogen storage materials can be divided into two periods, steady from 2011 to 2015 with a slight increase in 2013, with ups and downs during 2016–2021 and a visible decrease in 2020. Three main substance classes such as alloys, general inorganic, and small organic/inorganic molecules continued to be a focus of hydrogen storage research. During the 2011–2015 period, publications on alloys dominated as alloys were used for both physical storage (tanks/pipelines materials) and chemical storage (sorbents). It is interesting that research on polymers also increased at that time owing to polymer reinforcement of metal storage tanks. Growing interest in polymers may also have resulted from the application of porous polymers for hydrogen storage.^89,90^

Coordination compounds, which were of great interest in 2011–2015, show a decline in 2016–2021. There is continuous attention to hydrides with a surge in 2021. Elements and oxides are always in the spotlight. The most cited key substances for hydrogen storage are summarized in Table 2.

**Table 2:** Key Substances in Hydrogen Storage Research

substance class	substance	REG #	storage type/feature	2021 publications	exemplary publication
alloys	LaNi_5_	12196-72-4	chemical/hydrogenation	13	(152−156)
	Mg_2_Ni	12057-65-7	chemical/hydrogenation	11	(157, 158)
	FeTi	1223-04-0	chemical/hydrogenation	13	(159−164)
	stainless steel	12597-68-1	physical/tank material	16	(82, 165)
hydrides	MgH_2_	7693-27-8	chemical/dehydrogenation	86	(107 and 166−169)
	LiH	7580-67-8	chemical/dehydrogenation	17	(102, 170)
	NaBH_4_	16940-66-2	chemical/dehydrogenation	23	(171−176)
	AlH_3_	7784-21-6	chemical/dehydrogenation	12	(177, 178)
	LiAlH_4_	16853-85-3	chemical/dehydrogenation	14	(179, 180)
	Mg(BH_4_)_2_	16903-37-0	chemical/dehydrogenation	13	(181, 182)
elements	carbon	7440-44-0	physical/sorbent	100	(183−186)
	graphene	1034343-98-0	physical/sorbent	60	(187−191)
	graphite	7782-42-5	physical/sorbent	16	(192, 193)
	nickel	7440-02-0	chemical/catalyst	54	(194−196)
small organics	9-ethylcarbazole	86-28-2	chemical/dehydrogenation	16	(197, 198)
	methylcyclohexane	108-87-2	chemical/dehydrogenation	15	(199−202)
	ammonia	7664-41-7	chemical/dehydrogenation	37	(203−207)
	ammonia borane	13774-81-7	chemical/dehydrogenation	13	(208−212)
small inorganics	UiO-66(Zr)	1072413-89-8	chemical/sorbent	4	(213−215)
	HKUST-1	222404-02-6	chemical/sorbent	4	(216−218)
coordination compounds	Zn-MOF-5	255367-66-9	chemical/sorbent	5	(218−220)
oxides	MgO	1309-48-4	chemical/catalyst	14	(221, 222)
	Nb_2_O_5_	1313-96-8	chemical/catalyst	5	(223−225)
polymers	poly(ethylene glycol)	25322-68-3	chemical/dispersant for dehydrogenation catalysts	14	(226, 227)
	nylon-6	25038-54-4	physical/storage tank reinforcement	10	(228, 229)

The elements class includes carbon-based sorbents such as activated carbon (AC), carbon nanotubes (CNTs), graphitic nanofibers, graphene, graphite, and fullerenes as well as noble and transition metals which can be used as dehydrogenation catalysts and modifiers. Carbonaceous sorbents are promising materials for hydrogen storage owing to their low densities, good chemical stability, high surface area, and porosity.^91^ Research on these materials focuses on increasing the effective adsorption temperature by increasing their hydrogen binding energies, as well as improving volumetric and gravimetric storage capacities through optimizing the material’s porosity and surface area while studying the effects of material densification. The best results were achieved with carbon nanotubes.^92^ Carbon nanotubes, when decorated with metal or metal oxide nanoparticles, show a significantly improved hydrogen storage capacity. For instance, multiwalled carbon nanotubes (MWCNTs) decorated by Dy_3_Fe_5_O_12_ nanoparticles can store H_2_ at temperatures as low as −196 °C and pressures as low as 60 bar, providing a gravimetric density of 10.8 wt % and volumetric density of 41 kg/m^3^.^93^ The functionalization of ACs can result in reducing specific surface area due to pore blocking by metal nanoparticles, resulting in less H_2_ absorption. It was shown that the optimal metal loading is important to provide an appropriate hydrogen uptake by Ni-doped CNTs.^94^

Fullerenes are potential hydrogen storage materials that can react with hydrogen via the hydrogenation of carbon–carbon double bonds. Theoretically, 60 hydrogen atoms can be attached to the C_60_ fullerene spherical surface forming a stable C_60_H_60_ isomer with a hydrogen content of ∼7.7 wt %. The C_60_ hydrogenation reaction is reversible at high temperatures, about 550–600 °C.^95^ A new trend in carbonaceous sorbents is the preparation of porous carbon materials from biomass pyrolysis. It has been shown that the pyrolysis temperature, pyrolysis heating rate, and carbon-containing precursors strongly affect the yield and structure of the resulting porous carbons.^96^ Another attempt is to fabricate highly porous carbon sorbents by carbonizing highly crystalline metal–organic frameworks (MOFs) without any carbon precursors.^97^

Other widely used elements are transition metals which can be applied alone or together with noble metals (Pt, Pd) as dehydrogenation catalysts for metal hydrides and liquid organic hydrogen carriers. It was shown that using nickel nanoparticles as ammonia borane dehydrogenation catalysts is a promising step toward a feasible hydrogen storage medium for fuel cells.^98^

An example of common coordination compounds is porous MOFs, where H_2_ is physisorbed on the surface of the pores. In general, the “H_2_–MOF” interactions are very weak. Therefore, the high storage capacities of MOFs can be achieved at liquid nitrogen temperature and high pressures. Still, different MOFs have been constructed and extensively studied as potential hydrogen storage materials utilizing various metal ions such as Zn^2+^, Cu^2+^, Mn^2+^, Cr^3+^, and La^3+^ and ligands such as carboxylates, imidazolates, triazolates, and tetrazolates. Promising H_2_ storage data were reported for MOF-5 (4.5 wt % at −197 °C and 1 bar), prepared from benzene-1,4-dicarboxylate (BDC) and Zn^2+^ salt.^97^ The hydrogen release capacity of MOF-5 (41 kg/m^3^) is comparable with that of the activated carbon MSC-30 (38 kg/m^3^).^99^ Significant research is focused on densified MOFs including hybrid MOF@CNT and MOF/fullerene composites for hydrogen storage. Various MOFs such as MOF-5, MIL-101, Zr-MOFs, and HKUST-171 have been compacted and their hydrogen adsorption capacities evaluated.^100^ Compaction of UiO-66 at ∼7000 bar produced densified pellets capable of a total H_2_ uptake of 5.1 wt % at 100 bar and −197 °C compared to 5.0 wt % for the UiO-66 powder.^101^ Despite improving volumetric and gravimetric hydrogen absorption in MOFs, activated carbons showing similar performance are preferable because they are less expensive.

Light and complex metal hydrides are the most technologically relevant hydrogen storage materials as they require comparatively feasible working temperatures and have good hydrogen storage capacity. Research on metal hydrides focuses on improving the volumetric and gravimetric capacities, hydrogen adsorption/desorption kinetics, cycle life, and reaction thermodynamics of potential material candidates. To form light metal hydrides, hydrogen interacts with metals through different bonds. Ionic or covalent hydrides, such as LiH and MgH_2_, are quite suitable for hydrogen storage owing to their high hydrogen storage capacity but require a very high operating temperature. It was shown that noble metals (Pt, Ag, Au, Pd, and Ru) are beneficial to improve the dynamical stability and dehydrogenation properties of LiH.^102^ Although pristine MgH_2_ can store ∼7.6 wt % of hydrogen, both its hydrogenation and dehydrogenation reactions are very slow.^103^ However, the modification of MgH_2_ with various transition metal nanoparticles (Fe, Co, Ni, etc.) provides an additional hydrogen sorption mechanism via its active surface sites and thus improved kinetics.^104^ The H_2_ sorption by MgH_2_ has been enhanced after its doping with Fe and Ti nanoparticles supported on carbon nanotubes.^105^ Ball milling is now widely used for facilitating the hydrogenation/dehydrogenation process in magnesium-based hydrogen storage materials. The mechanical milling helps to pulverize the particles of MgH_2_ into micro- or nanocrystalline phases and thus leads to lowering of the activation energy of desorption.^106^ The activation energy can be drastically lowered by doping the milled MgH_2_ with nanocatalysts as a result of increasing the collision frequency between H_2_ molecules and nanoparticles by reducing the crystallite size.^107^

**Figure 9:**
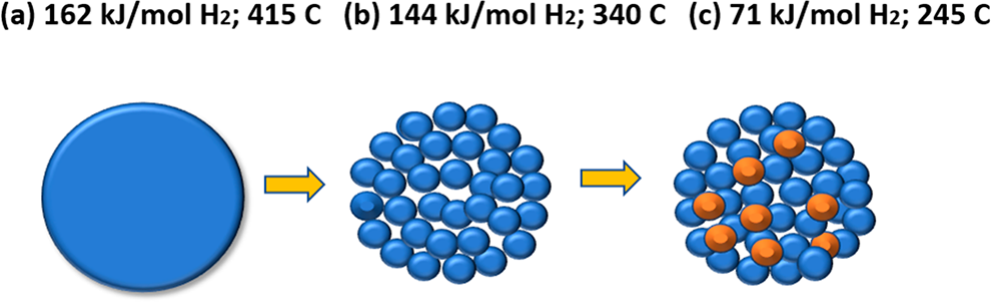
Activation energies of H_2_ sorption and dehydrogenation temperatures for MgH_2_ (a) bulk, (b) ball-milled nanocrystalline, and (c) nanocrystalline transition metal nanoparticle-doped MgH_2_.

The activation energies of the H_2_ sorption and dehydrogenation temperatures for the bulk MgH_2_, mechanically milled MgH_2_, and milled nanocatalyst-doped MgH_2_ are shown in Figure 9. Hydrogen release occurs at 415, 340, and 245 °C for commercial, ball-milled nanocrystalline, and nanocrystalline nanocatalyst-doped MgH_2_, respectively.

Both alkali- and alkaline-earth metals borohydrides have a significant hydrogen storage capacity. For example, LiBH_4_ demonstrates the highest gravimetric (18.5 wt % H_2_) and volumetric (121 kgH_2_/m^3^) hydrogen storage capacity.^108^

Sodium borohydride (NaBH_4_) is an excellent hydrogen storage material. To regenerate NaBH_4_ with high yield and low costs, the hydrolytic product NaBO_2_ reacts with CO_2_, forming Na_2_B_4_O_7_·10H_2_O and Na_2_CO_3_, both of which are then ball-milled with Mg under ambient conditions to form NaBH_4_ in high yield (close to 80%).^109^ This method is expected to effectively close the loop of NaBH_4_ regeneration and hydrolysis, enabling a wide deployment of NaBH_4_ for hydrogen storage.

Hydrogen storage alloys are designed to absorb and release hydrogen without compromising their own structure. In general, they use magnesium, rare-earth, and titanium to store hydrogen by reacting with it to form hydrides.^110^ Magnesium-based alloys such as Mg_2_Ni have the most promising hydrogen storage properties; intermetallic compounds such as TiFe and LaNi_5_ have good hydrogen storage reversibility.^111^ LaNi_5_-based alloys have been used in practical applications because of their stable reversible hydrogen absorption and desorption reactions under moderate conditions (i.e., room temperature and hydrogen gas pressure less than 10 bar).^112^ Recently, a new promising class of H_2_ storage alloys, TiZrNbFeNi high-entropy alloys (HEAs), has been discovered.^113^ The presence of lattice strain in the HEA distorted crystal structure can be beneficial to store hydrogen. Recent studies of rare-earth/magnesium/nickel alloys, RE_(2–*x*)_Mg_*x*_Ni_4_, showed that reversible hydrogen absorption and desorption can be controlled by selecting an appropriate RE element and RE/Mg ratio.^114^ Hydrogen storage alloys represent an excellent solution for fuel cell storage.

Small inorganic/organic molecules are widely used as hydrogen storage materials. Ammonia is a potential carrier capable of converting hydrogen into liquid fuel, making it a beneficial form of long-term hydrogen storage. Ammonia’s energy density by volume (12.7 GJ/m^3^) is higher than that of liquefied hydrogen (8.5 GJ/m^3^) and compressed hydrogen (4.5 GJ/m^3^), making it far easier to store and transport.^115^

Ammonia borane (AB), BH_3_NH_3_, is a stable solid at room temperature, melting at 110–114 °C, which makes it a promising hydrogen storage material for use in fuel cells for the automotive industry. The only technical barrier of storing H_2_ on-board in the form of solid AB is typical of any solid fuel that needs to be regenerated.^86^

Formic acid, with its high volumetric concentration of H_2_ (53 kg/m^3^), low toxicity, and biodegradability, is a promising renewable hydrogen carrier.^116^ It can be produced from carbon dioxide via direct catalytic hydrogenation. To improve hydrogen generation by FA selective dehydrogenation, highly efficient catalysts based on noble metals such as ruthenium and iridium are required.^116^

*N*-Alkylcarbazoles are liquid organic H_2_ storage materials that require dehydrogenation catalysts. Developing a catalyst with higher conversion, better selectivity, and stability is a current bottleneck of *N*-alkylcarbazole dehydrogenation technology. Binuclear ruthenium and rhodium catalysts,^117^ noble metal catalysts,^118^ and Ru–Ni/TiO_2_-supported catalysts^119^ have been used for *N*-ethylcarbazole dehydrogenation. Recently, the use of nickel in bimetallic composites with Pd or Ru has been reported.^110^

General inorganics in hydrogen storage mostly include transition metal carbides, nitrides, and phosphides as hydrogenation/dehydrogenation catalysts and modifiers.

Two-dimensional MXenes, such as Ti_3_C_2_, Ni_3_C, Mo_2_C, Cr_3_C_2,_ and NbC, have been used to enhance the H_2_ storage behavior of MgH_2_.^120^ The carbides were introduced into MgH_2_ by mechanical ball milling without changing their phase. Ti_3_C_2_ shows the best catalytic effect on MgH_2_ dehydrogenation kinetics, followed by Ni_3_C, NbC, Mo_2_C, and Cr_3_C_2_.^120^ Doping with nickel, Ni/Ti_3_C_2_, improves the catalytic properties of Ti_3_C_2_ toward the dehydrogenation of metal hydrides.^121^ A recent study on hydrogen sorption by 2D tin carbide monolayers decorated with alkali metals, AM-2D SnC (AM = Li, Na, and K), showed that the K-2D SnC monolayer has the highest hydrogen storage capacity with one K atom adsorbing up to six H_2_ molecules, followed by Na-2D SnC with five H_2_ molecules and Li-2D SnC with three H_2_ molecules.^122^ These complexes can potentially reach the US-DOE recommended target of 5.5 wt % for on-board automotive systems.

Theoretical studies of hydrogen adsorption on the pristine bilayer hexagonal boron nitride (h-BN) show a trend of decreasing binding energies and desorption temperatures that is useful for potential H_2_ storage. The calculated overall storage capacity of the h-BN is 6.7 wt % with *E*_ads_ of 0.223 eV/H_2_.^123^ Studies of heat treatment effects on TiO_2_-coated boron nitride nanofibers revealed that the highest hydrogen adsorption occurred at room temperature.^124^

Recently, aluminum nitride nanoclusters have been investigated for hydrogen storage. It has been shown that alkali-earth metal (Be, Mg, and Ca)-encapsulated Al_12_N_12_ nanoclusters demonstrate an increase in the H_2_ adsorption energy and a decrease in the HOMO–LUMO energy gap when compared to untreated Al_12_N_12_ and H_2_–Al_12_N_12_.^125^ Another example of nanomaterials for hydrogen storage is a graphitic carbon nitride, g-C_3_N_4_, whose structure permits the storage of a significant amount of hydrogen.^126^

A DFT study on the thermodynamic stabilities of Li- and Na-decorated 2D boron phosphide (BP) monolayers suggested that BP can serve as an effective H_2_ storage material. The 2D BP surface modification with Li or Na atoms significantly increases both the H_2_ binding energies and the H_2_ storage capacities.^127^ An improvement in hydrogen storage capacity due to the process of intracell Kubas-enhanced hydrogen adsorption in Co_2_P nanoparticles obtained by ball milling has also been discovered.^128^

Metal oxides are promising candidates for long-term hydrogen storage. They participate in a reversible redox cycle using water steam as an oxidizing agent and H_2_ as a reducing agent. The oxidation reaction results in a high-purity hydrogen production (eq 10).^129^
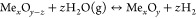
10

The best oxides for H_2_ storage are found to be Fe_3_O_4_, GeO_2_, MoO_2_, SnO_2_, ZnO, and WO_3_ supported on Al_2_O_3_, TiO_2_, Cr_2_O_3_, MnO, and MgO.^129^ Ti_4_M_2_O_*y*_ mixed oxides (Ti_4_Fe_2_O, Ti_4_Ni_2_O) have demonstrated good hydrogen storage properties at room temperature.^130^ Also, hybrid ceramics, such as NiCo_2_O_4_/TiO_2_, are efficient and novel hydrogen storage materials.^131^ Transition metal oxides are universally used as hydrogenation and/or dehydrogenation catalysts. For example, TiO_2_ supported on MWCNTs improves both hydrogenation and dehydrogenation of a Mg–Ni alloy (absorbs 5.60 wt % H_2_ at 99.9 °C and releases 6.08 wt % H_2_ at 280 °C).^132^ The addition of TiO_2_ to a Mg_80_Ni_10_La_7_Ce_3_ alloy significantly improves its dehydrogenation.^133^ The nanoparticles of metal oxides are highly efficient catalysts when it comes to hydrogenation/dehydrogenation. Thus, Ni@TiO_2_ core–shell nanoparticles significantly improve hydrogen desorption from MgH_2_.^134^ The catalytic effect of milled nanocrystalline VNbO_5_ on MgH_2_ dehydrogenation is also remarkable.^135^ Recently, solid oxides with ABO_3_ perovskite structures have been frequently mentioned because they have enhanced hydrogen storage properties.^136^ It was shown that porous NiTiO_3_ and CoTiO_3_ nanorods can decrease the dehydrogenation temperature of MgH_2_ and provide faster hydrogen desorption (*T*_des_ = 261.5 and 298 °C for NiTiO_3_ and CoTiO_3_, respectively).^137^ The scheelite-ABO_4_ crystal structure oxides such as NiMoO_4_ and CoMoO_4_ nanorods similarly enhance the nonisothermal and isothermal desorption performance of magnesium hydride.^138^

#### Polymers

Porous organic polymers can store and release hydrogen through hydrogen physisorption on their highly porous structures and in some cases use combinations of physisorption and chemisorption to store H_2_. Some examples are hyper-cross-linked polymers (HCPs), polymers of intrinsic microporosity, conjugated microporous polymers, and porous aromatic frameworks.^139^ The absorption of hydrogen by the polymers is strongly determined by their specific surface area and porosity. The larger the surface area and the smaller the pore size, the greater the amount of H_2_ absorbed.^140^ HCPs synthesized via the Friedel–Crafts method are particularly attractive for H_2_ storage owing to their large internal surface area, high microporosity, and thermal and chemical stability.^141^ However, it was recently demonstrated that H_2_ absorption at 14 MPa results in irreversible deformations of HCP and HCP composites with graphene oxide (GO).^142^ Thus, HCPs and HCP–GO composites cannot be used for high-pressure H_2_ absorption.

The introduction of functional groups in polymer networks can also provide an enhancement of the hydrogen uptake. Porous polymers containing highly electron-deficient carboranes were successfully used as hydrogen sorbents.^143^ Ketone- and *N*-heterocycle-containing polymers can fix and store hydrogen at atmospheric pressure through the formation of the corresponding alcohol and hydrogenated *N*-heterocycle polymers, respectively. The hydrogenated polymers will release hydrogen in the presence of catalysts at mild conditions. Thus, a quinaldine-substituted poly(acrylic acid) and its hydrogenated 1,2,3,4-tetrahydroquinaldine derivative reversibly release hydrogen when heated at 80 °C in the presence of an aqueous iridium complex catalyst.^144^ Conducting polymers consisting of a polyaniline matrix, which can be functionalized by catalytic doping or by the introduction of chemical groups into a polymer molecule, are promising candidates for hydrogen storage. It was reported that polyaniline could store up to 6–8 wt % of hydrogen.^145^ Porous polyaniline P-PANI facilitates the hydrogen diffusion and reaction kinetics of the hydrogen storage alloys.^146,147^ Polyacrylamide blending with ammonia borane enables the dehydrogenation of the polymeric composite to occur at a lower temperature with enhanced hydrogen purity.^148^ Although hydrogen storage by means of physisorption has some limitations, polymers seem to be very promising materials, owing to their high potential for structural and functional tuning, as well as good thermal and chemical stability.

### Nanomaterials for H_2_ Storage

Nanomaterials for hydrogen storage have attracted great interest in recent years. As shown in Figure 4, “nanoparticles” are the most popular concept followed by “nanocomposites” and “nanostructures”. Metal hydride nanoparticles and polymer and metal–organic frameworks nanocomposites are advantageous for storing substantial amounts of hydrogen.^149^ Carbon “nanotubes” are efficient H_2_ storage materials. “Nanocrystals” and “nanocatalysts” concepts reflect the synergistic effects of nanocrystallinity and nanocatalyst doping on improving the thermodynamics and hydrogen reaction kinetics in metal hydrides. The development of new nanocatalysts maximizes the hydrogenation/dehydrogenation efficiency while minimizing the use of precious noble metals.^150^ “Nanoporous materials” confirm that the nanoconfinement of hydrides and borohydrides in carbon nanopores significantly improves their hydrogen sorption properties.^151^ The nanoconcepts extracted from 2011–2021 publications confirm the widespread use of nanostructured materials in hydrogen storage.

**Figure 10:**
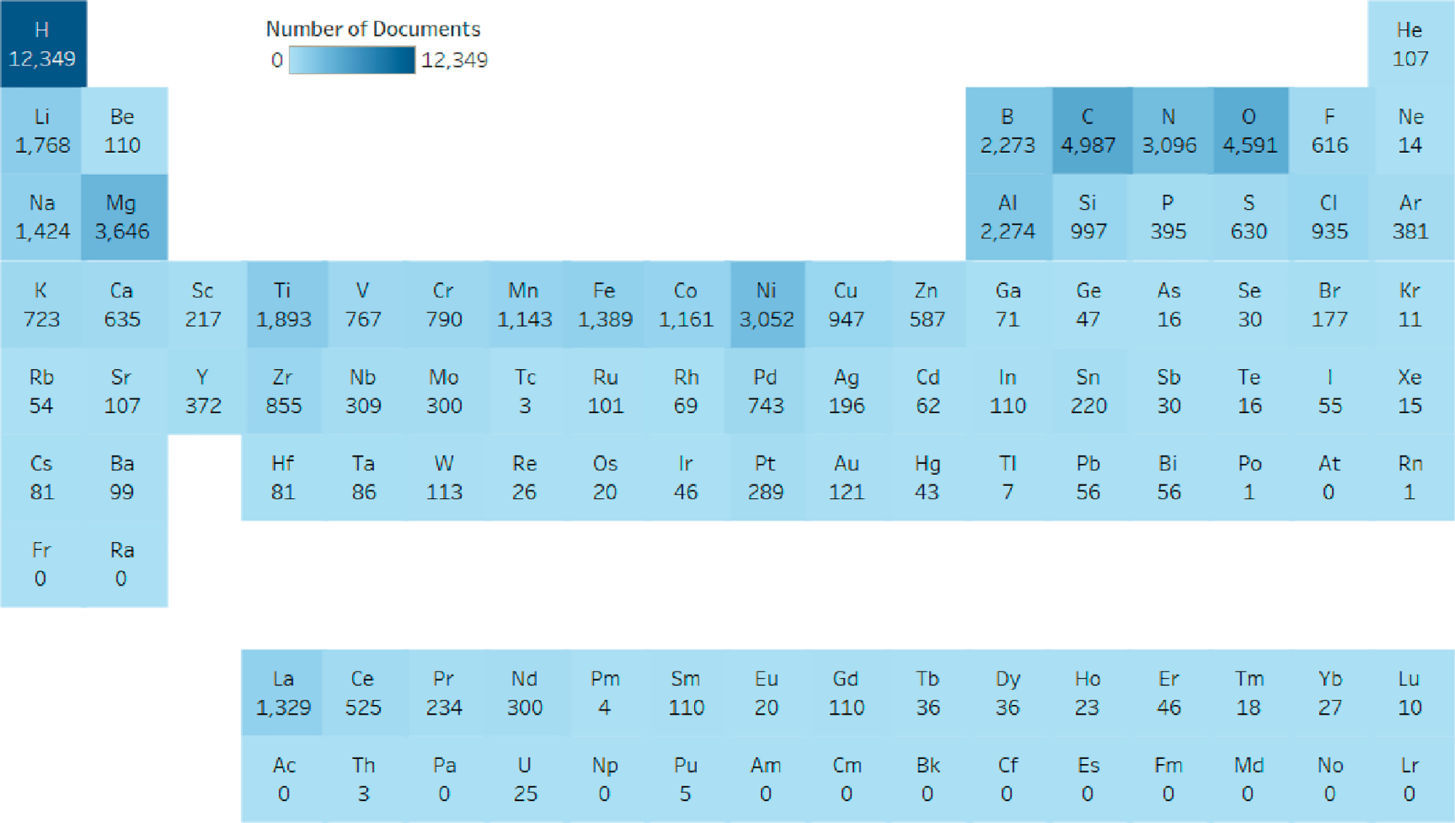
Occurrence of elements in materials used for hydrogen storage research by number of documents from 2011 to 2021.

As discussed above, carbon is the most prevalent element in H_2_ storage (Figure 10). Carbon is a major part of various carbonaceous sorbents such as activated carbon, graphene, MOFs, and liquid organic hydrogen carriers and polymers. Magnesium is another key element found in hydrides, borohydrides, and hydrogen storage alloys (MgH_2,_ Mg(BH_4_)_2,_ and Mg_2_Ni). Transition metals, namely Ni, La, Ti, and Fe, are important components of H_2_ storage alloys (LaNi_5_, FeTi) and metal oxides (Ti_4_Fe_2_O, Ti_4_Ni_2_O). In addition, the growing interest in transition metal nanoparticles as dehydrogenation catalysts derives from the fact that they can successfully replace expensive noble metals.

## Hydrogen Utilization

### Utilization of Hydrogen to Generate Energy

With hydrogen now generated and stored for future use, we now concentrate on methods of converting the hydrogen into energy. The primary method of using hydrogen in the GHE is a fuel cell, an electrochemical device that converts chemical energy into electrical energy. While both batteries and fuel cells convert energy released in chemical reactions to electrical energy, fuel can be supplied continuously to fuel cells, which allows fuel cells to provide uninterrupted electrical energy.^230^ A diverse range of applications have already been commercialized, such as cars, stationary power generation, portable military equipment, and even toys.^231^

**Figure 11:**
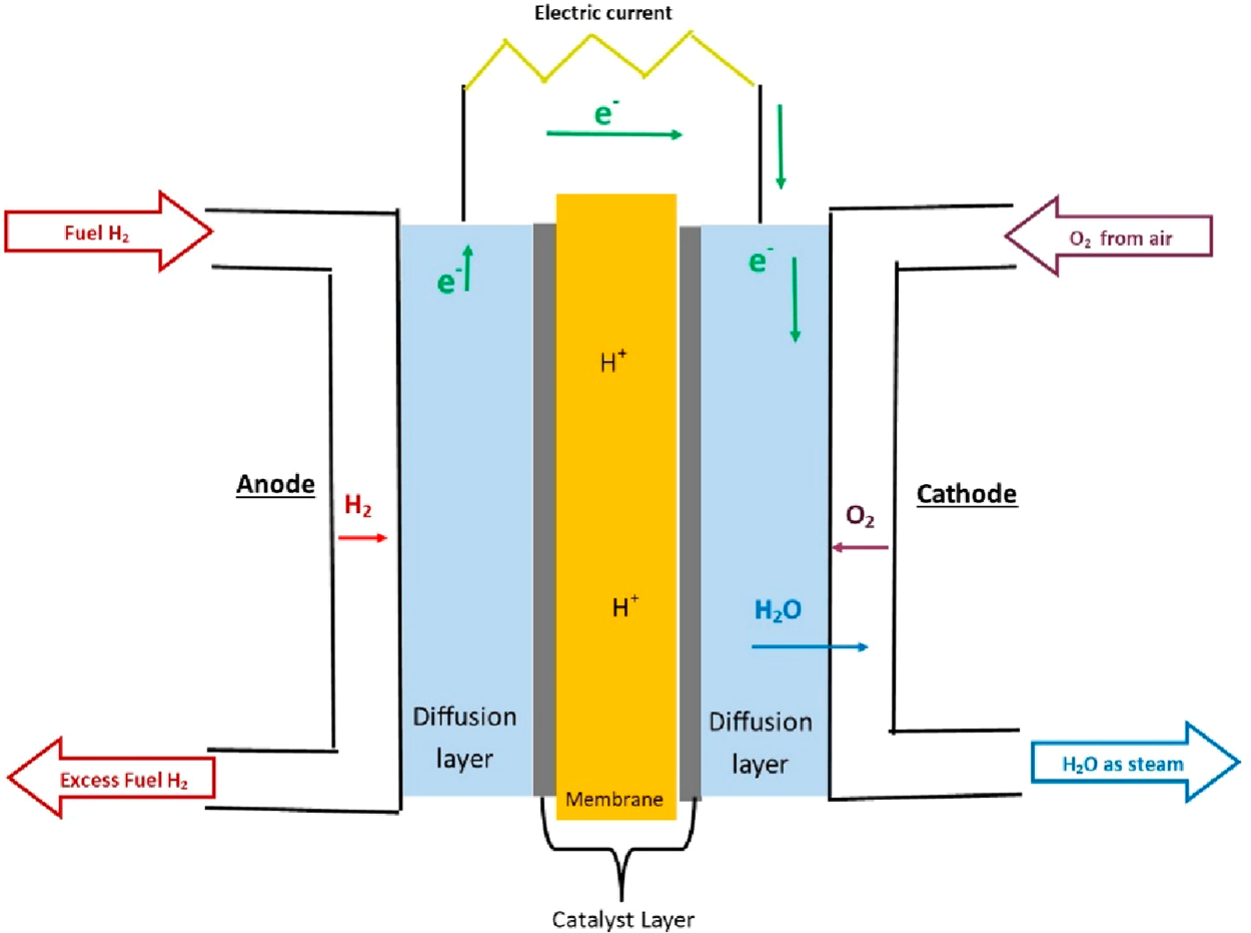
General structure and operation of a hydrogen fuel cell.

A fuel cell that uses hydrogen fuel works through a process that is the reverse of the before-mentioned water electrolysis. Instead of using water and electricity to produce hydrogen and oxygen, fuel cells use hydrogen fuel and oxygen from the air to produce water, usually as steam. In general, a typical fuel cell consists of a thin electrolyte material, typically a semipermeable membrane, in between two porous electrodes, the cathode and anode.^232^ Molecular hydrogen is delivered to the anode via a gas flow, where the anode catalyst oxidizes the hydrogen, producing hydrogen cations and electrons. This reaction is called the hydrogen oxidation reaction (HOR). The hydrogen cations pass through the electrolyte/membrane from the anode to the cathode. The electrons in the system cannot transfer from the anode to the cathode through the layers of the electrolyte but only through an external electrical circuit.^230^ It is this movement of electrons that produces the electric current. At the cathode, molecular oxygen combines with the hydrogen protons and electrons to form water. This reaction is called the oxygen reduction reaction (ORR) and is the limiting reaction in a fuel cell owing to its slow kinetics. Figure 11 shows this process in a single unit cell.

Hydrogen fuel cells work on the principle of three partial reactions:
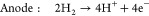
11
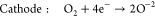
12
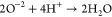
13
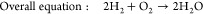
14

Catalysts that are strong enough to absorb H_2_ and/or O_2_ to break the molecular bond, weak enough to release the resulting product, selective enough to minimize unwanted side products, and stable or inert enough to withstand the operating fuel cell environment are needed for these reactions to proceed in a fast and efficient manner.^233^ In general, platinum or platinum-based catalysts are the ideal catalysts for both ORR and HOR for they satisfy all previously mentioned requirements. Just like with the case of electrolyzers, owing to the high cost of Pt, extensive research into replacing this noble metal or lowering its loading use to lower fuel cell costs is ongoing (Table 3).

**Table 3:** Key Substances in Hydrogen Fuel Cell Research

substance class	substance	REG #	publications	feature/areas of interest	exemplary publication
alloys	cobalt platinum alloy	11134-15-9	42	catalyst; focus on reducing the cost of ORR catalysts by reducing Pt via nanostructuring, catalyst ink formulations, alloying with non-noble metals, etc.	(251, 252, 256, 259, and 265)
	platinum alloys	1273120-99-2	53	catalyst; reducing the cost of ORR catalysts by reducing Pt via high surface area nanoalloys/Pt–M nanoparticles	(254, 258)
elements	carbon	7440-44-0	1562	catalyst support; alternatives to noble metal catalysts for ORR via non-noble metal–N–C catalysts, high surface area micro/nanostructures of Pt/C and non-Pt catalysts, increase of surface defects and porous structures	(253, 255, 257, 266, and 267)
	graphene	1034343-98-0	461	catalyst support; filler material for PEMFCs; alternatives to noble metal catalysts for ORR via doping of graphene structure with silicon, sulfur, and/or nitrogen usually on non-noble metal graphene electrocatalysts	(255, 264, and 268−270)
	nickel	7440-02-0	694	electrode/electrolyte component SOFCs; ORR and/or HOR catalyst with focus on nanostructures, porosity, single-atom alloys, and nano/microstructures; metal foam as a flow distributor in PEMFCs; attempts to reduce Pt via nanoalloys of Pt as catalysts	(258 and 271−277)
	platinum	7440-06-4	1239	most used and versatile catalyst component, expensive, focus on reducing loading amount of Pt via nanoalloys, micro/nanostructures, and Pt–M catalysts	(266 and 278−280)
oxides	CeO_2_	1306-38-3	250	interlayer material between electrode and electrolizer in ceramic fuel cells; doped ceria catalyst/electrolyte for SOFCs; used in interfaces of membrane and catalysts in PEMFCs to better contact area	(276 and 281−284)
	SiO_2_	7631-86-9	221	used as a template for catalyst synthesis; component in proton exchange composite membranes; hybrid nanofluid coolant for PEMFCs	(285−288)
	TiO_2_	13463-67-7	315	ORR catalyst nanocomposite component; catalyst support; organic–inorganic composite membranes for AEMFCs	(289−292)
	NiO	1313-99-1	225	part of ceramic anode or cathode composition for SOFCs, usually reduced to Ni	(274, 293, and 294)
	Y_2_O_3_	1314-36-9	127	SOFC solid electrolyte dopant or electrode component, focus on formation techniques (printing, plasma spray); degradation studies and microstructures	(295−298)
	Y_*x*_Zr_*x*_O_*x*_	64417-98-7	125	SOFC electrolyte or electrode component, focus on perovskite structure modification, electrode–electrolyte interface, and degradation	(282, 297, and 299)
	ZrO_2_	1314-23-4	210	SOFC electrolyte; focus on replacing with materials that lower operating temp, obtaining electrolyte thin layer structures, degradation studies, and microstructures	(297, 300, and 301)
polymers	ethene, homopolymer	9002-88-4	83	bettering performance and durability of proton-conducting membranes; polyethylene-based anion-exchange membranes	(243, 302, and 303)
	poly(vinylidene fluoride)	24937-79-9	103	proton-conducting membranes; functionalization for selective proton conducting, polymer–ceramic composites for SOFCs	(241, 304)
	polypropylene	9003-07-0	75	anion exchange membranes, functionalization for better conductivity; conductive polymer composites for bipolar plates	(263, 305, and 306)
	poly(tetrafluoroethylene)	9002-84-0	304	enhanced proton-conducting membranes; support/sublayers for catalysts; interest in porosity and better PTFE loading	(242 and 307−309)

Fuel cells in general can be categorized by various criteria such as the type of fuel or operating temperature, but because this paper will be focused on hydrogen fuel cells, we will classify them according to the type of electrolyte they employ. The four types of fuel cells that tend to use hydrogen as their primary source of fuel are alkaline fuel cells (AFCs), proton exchange or polymer electrolyte membrane fuel cells (PEMFCs), phosphoric acid fuel cells (PAFCs), and solid oxide fuel cells (SOFCs).

Alkaline fuel cells were first widely used in the U.S. space program to produce electrical energy and water on-board a spacecraft, specifically in the Apollo missions to the Moon and later in the space shuttle program, specifically in the orbiter.^234,235^ AFCs have also been used on vehicles like forklifts, as stationary power applications, as backup power, and in military applications.^235,236^ The electrolyte is a concentrated alkaline solution, usually potassium hydroxide owing to its high conductivity, and operates on average below 100 °C, though temperatures can range from below zero to 230 °C.^231,235,236^ Anion exchange membranes have also been used as electrolytes.^235^ AFCs differ from other fuel cells because the electrolyte/membrane conducts hydroxyl anions (OH^–^) instead of H^+^ cations.^235^ The reactions that take place at each electrode are the following:
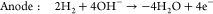
15
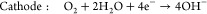
16
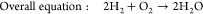
17

The alkaline medium causes the ORR to have faster kinetics and allows greater material compatibilities, which in turn permit the use of a wide range of electrocatalysts other than platinum to be used. For example, high surface area nickel doped with Ti, Cr, La, or Cu to prevent its oxidation is a cheap and active alternative catalyst for the anode, while some alternative catalysts for the cathode (that are more affordable than platinum while having good O_2_ reduction catalytic activity) are pyrolyzed macrocycles on a carbon support, manganese oxides, perovskite-type oxides, and MnCo_2_O_4_.^234^

The biggest disadvantage of AFCs is their high sensitivity to contaminants, especially CO_2_, which reacts with KOH to form K_2_CO_3_, degrading the cell performance and durability. CO_2_ poisoning has required pure hydrogen and oxygen to be used instead of air or for the CO_2_ to be removed. It can be removed via absorbers (soda lime or molecular sieves) or by electrolyte recirculation where the electrolyte is passed through a cleaning system to remove carbonates.^237^ Using an alkaline membrane as a portion of the electrolyte helps, reducing the susceptibility of CO_2_ poisoning. However, carbon dioxide still affects the performance of the alkaline membrane fuel cells (AMFCs).^238^

Proton exchange membrane fuel cells were invented by General Electric for use in NASA’s “Gemini” manned space vehicles.^239^ Also known as solid polymer electrolyte membrane fuel cells, they use a solid, acidic polymer membrane that conducts hydrogen cations through its structure when saturated with water. Most commercial cells use a perfluorosulfonic acid ionomer membrane from the Nafion family developed by DuPont.^240^ A primary advantage of PEM cells is their low weight in comparison to liquid electrolytes, making them the main fuel cell candidates to power electric vehicles as well as more portable power applications, although stationary applications are also possible.^231,238−240^

The disadvantage of PEM cells is that the membrane must be hydrated to conduct protons, which means the membrane must be kept at around 80 °C, below the boiling point of water (though high-temperature versions above 200 °C have been studied), and water management in general can be an issue.^231,239,240^ Research on the sulfur functionalization of polymer membranes or on new nanocomposite polymer membranes shows promising results, allowing for the membrane to need less water saturation and for PEMFCs to run at higher temperatures with enhanced ion conductivity.^241−243^ PEM fuel cells also require very pure hydrogen with minimal or no CO, which poisons the expensive platinum catalysts at low temperatures, but this issue can be avoided if hydrogen from water electrolysis is used instead of hydrogen produced by steam reforming. PEMFCs have the same operating principles shown in Figure 10 and eqs 11–14.

Phosphoric acid fuel cells use phosphoric acid (H_3_PO_4_) in silicon carbide as the electrolyte. This acid is a solid at room temperature but melts at 42 °C and is stable at 200 °C, allowing this cell to operate at higher temperatures and reducing the sensitivity to carbon monoxide poisoning.^244^ The advantage of this high operational temperature is that not only can these fuel cells be used in stationary power applications, but also the waste heat from operation can be captured and used for space heating and hot water.^244^ The disadvantages of the cell are that it must be heated first for it to be able to operate (long startup), that because of its high acidity and operating temperatures it usually uses pure platinum as the catalyst for the cathode and a platinum–ruthenium alloy for the anode (ruthenium helps reduce CO poisoning), and that it is very sensitive to sulfur contaminants.^236,244^ Its operating principles are the same as those for PEMFCs.

Solid oxide fuel cells use an insulating ceramic solid oxide, most commonly ZrO_2_ doped with Y_2_O_3_ (yttria-stabilized zirconia), as the electrolyte that conducts oxygen ions.^245^ Owing to the solid structure of the electrolyte, it has a very high operating temperature between 600 and 1000 °C to achieve sufficient ionic conductivity.^246^ The high temperature of operation is a disadvantage owing to slow device startup and thermal shielding requirements, but it also removes the need for a precious-metal catalyst, thereby reducing cost.^238^ SOFCs are also the most sulfur-resistant fuel cell type and are not poisoned by carbon monoxide.^236^ High operating temperatures also cause durability issues and strict material requirements. Therefore, the development of high-temperature-stable device materials and lowering the temperature of the cells to more intermediate levels (400–700 °C), for example, using lower temperature oxides like bismuth vanadates^247^ and zinc oxides,^248^ are the main challenges facing this technology.^249^ These types of fuel cells are mostly used for stationary applications (auxiliary power, electric utility, and distributed generation) and are highly used for the production of electrical and useful thermal energy known as combined heat and power (CHP).^236,245^

### New Directions in Hydrogen Fuel Cells

**Figure 12:**
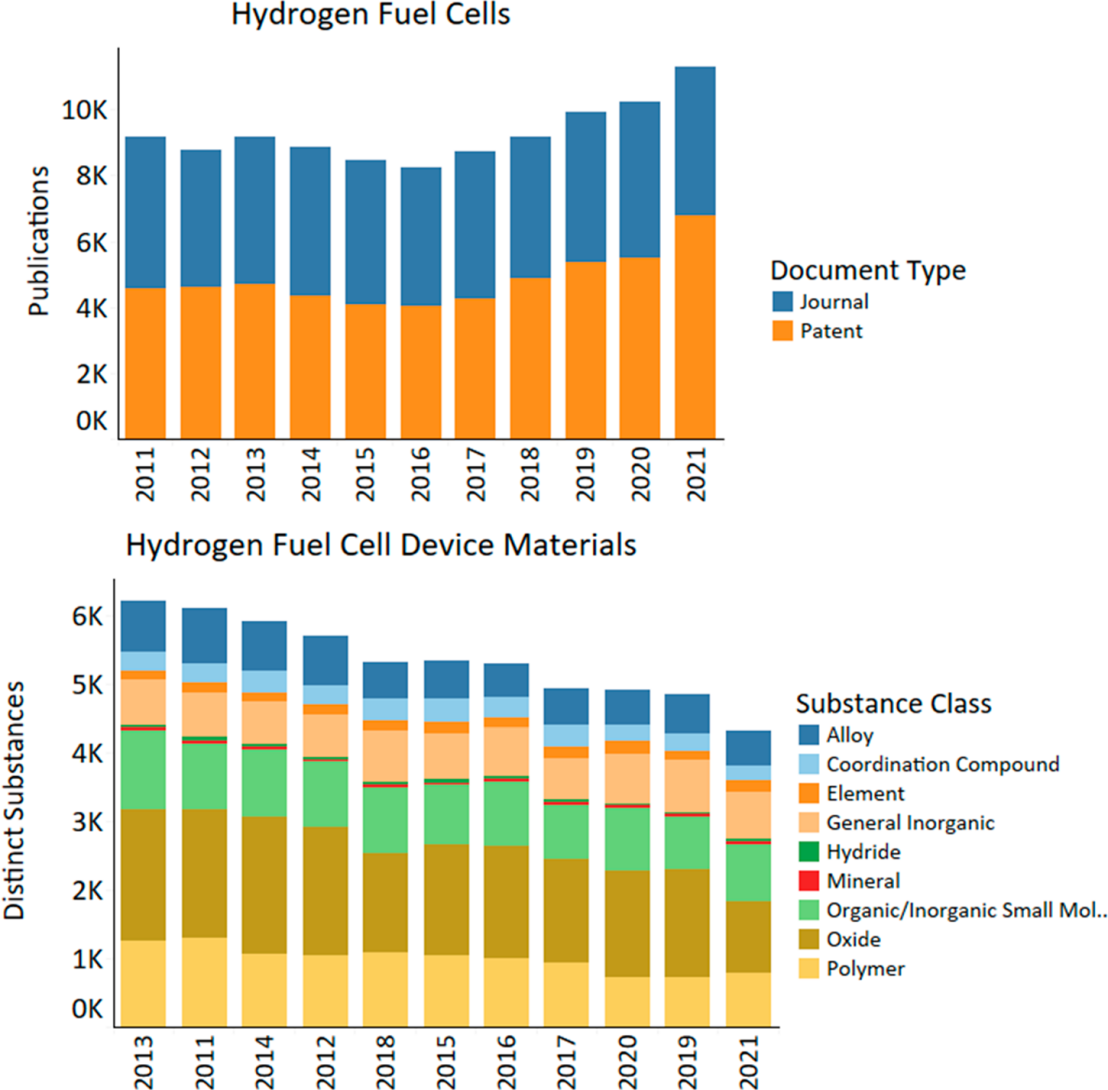
Publication trends and distinct substances used by year in hydrogen fuel cell research from 2011 to 2021.

In the area of hydrogen fuel cells, the publication volume was flat with an almost equal distribution of documents between patents and journals up to 2018 (Figure 12). It is in 2018 that one begins to see a rise in patents as well as documents in general, with 2021 being the year of the most overall interest and patent publications.

**Figure 13:**
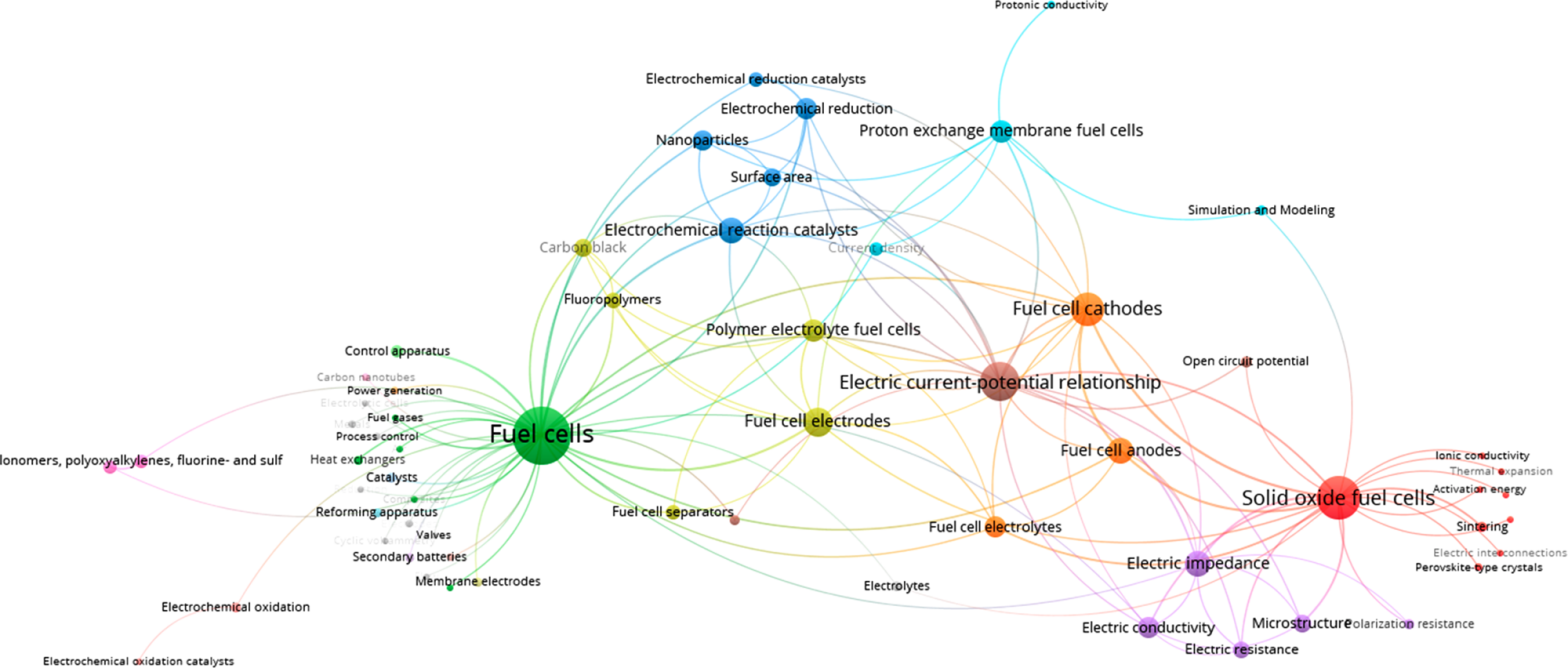
Top 125 pairs of co-occurring concepts in the hydrogen fuel cell literature from 2011 to 2021.

Similar to water splitting, the fuel cell field seeks to improve performance and durability while lowering the costs of fuel cell components to make it more viable for market applications.^231,250^ This is reflected in the most commonly co-occurring concepts clustered network diagram (Figure 13). The primary concept in the literature is, as expected, “fuel cells”, but it is important to note that it is followed by “solid oxide fuel cells”. SOFCs garnered more attention in the 2011–2021 decade than “proton exchange membrane fuel cells” or “polymer electrolyte fuel cells”. One can also see that there is no mention of “alkaline fuel cells” or “phosphoric acid fuel cells” in the top 125 concepts, implying that the research and development of SOFCs and PEMFCs is preferred.

Another popular concept is the “electric current–potential relationship”, a standard concept used when referring to the voltammetry measurements of the cell or half-cell performance utilized in performance evaluation, as can be seen in its link with the “impedance” cluster that includes impedance, current density, conductivity, etc. With regard to the individual fuel cell components, the data show that although “fuel cell electrodes” are a relatively popular topic, higher interest in “fuel cell cathodes” exists. We suspect this is due to research efforts directed toward the ORR, which occurs in the cathode of most fuel cells, being one of the major challenges when it comes to reducing cost.^251−259^ One can also see the interest in tackling this challenge with other concepts, particularly “electrochemical reaction catalysts” and “electrochemical reduction” clusters. Like water splitting, the inclusion of concepts such as “nanoparticles” (Figure 4) and “surface area” in this cluster confirms morphology and surface research as important parameters when optimizing catalysts. This is also supported by the key role of carbon and graphene in hydrogen fuel cell research (Table 3).

The diversity in materials used in hydrogen fuel cell devices in GHE research has slowly declined (Figure 12), with 2013 being the year with the highest number of distinct substances being researched. The major interest throughout the decade has been in oxides owing to the growing interest in materials for SOFCs. The constant appearance of organic/inorganic small molecules is mostly due to electrolyte components like water, acids as alternative electrolytes (e.g., phosphoric acid and sulfuric acid fuel cells), alternative oxidants like H_2_O_2_,^260^ and association with chemical hydrogen storage like ammonia and methanol.^205,206,261,262^ Continuous polymer interest is due to research on membranes for the PEMFC and AEMFC electrolytes.^242,243,263,264^

There has been some continued interest in substances under the alloys and elements designation owing to catalyst research. As before mentioned, the never-ending pursuit of more affordable yet effective catalysts is also reflected in the interest of nanoparticles (Figure 4). Just as in the case of green hydrogen production and hydrogen storage, nanoparticles and nanomaterials play a larger role in hydrogen fuel cell development. The application of these materials is diverse, but the literature shows that the main interests are for the increase of surface area or porosity or just general improvements in the morphology of fuel cell catalysts, especially for the ORR.^252,254,256,258,259^ This is further explored and demonstrated in the features of the most popular substances in hydrogen fuel cell research and their exemplary publications shown in Table 3.

**Figure 14:**
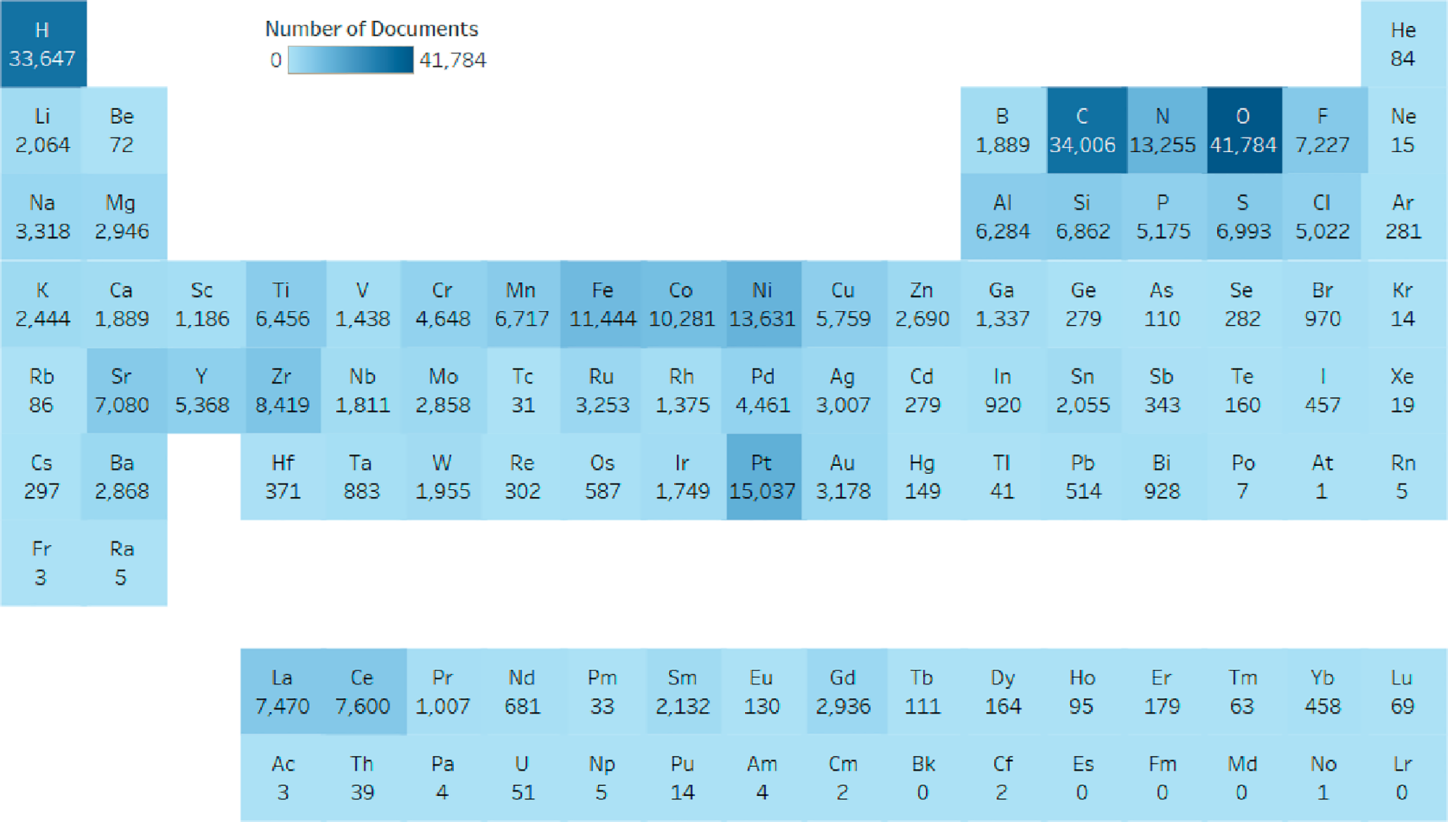
Occurrence of elements in materials used for hydrogen fuel cell device research by the number of documents from 2011 to 2021.

Many of the substances in Table 3 are composed of the highlighted elements in Figure 14. Some of the highlighted elements are part of the basic components of fuel cells, for example: hydrogen as fuel; oxygen for the ORR or oxides in electrodes; Pt, Ni, N, Fe, and C for catalysts, etc. However, Table 3 does not show all substances associated with solid oxide fuel cell electrolytes and electrodes, especially the important perovskite-like ceramics which are reflected in Figure 14 by the high occurrence of Co, La, and Sr.

Cobalt, lanthanum, and strontium are very common components in perovskites, a type of crystalline material. The compositions of perovskites are very varied, and modifications of the structure for better efficiency and durability are a major research topic in solid oxide fuel cells, especially because perovskites can be used as both electrodes and electrolytes in SOFCs.^246,282,310^ A great example that includes all three is the perovskite lanthanum strontium cobalt ferrite (LSCF), one of the leading materials for intermediate temperature SOFCs that possesses mixed ionic and electronic conductivity (MIEC).^311,312^ Though cobalt-containing cathodes are known to have good performance, there is also a lot of interest in cobalt-free cathodes owing to the high cost and compatibility issues with electrolytes.^313^ Many of these cobalt-free perovskites still contain La and Sr as well as other elements such as Fe, Ba, Cu, Ti, Cr, and Sm, among others, that are reflected in Figure 14.

## Global Publication Trends in GHE Technologies by Geography

**Figure 15:**
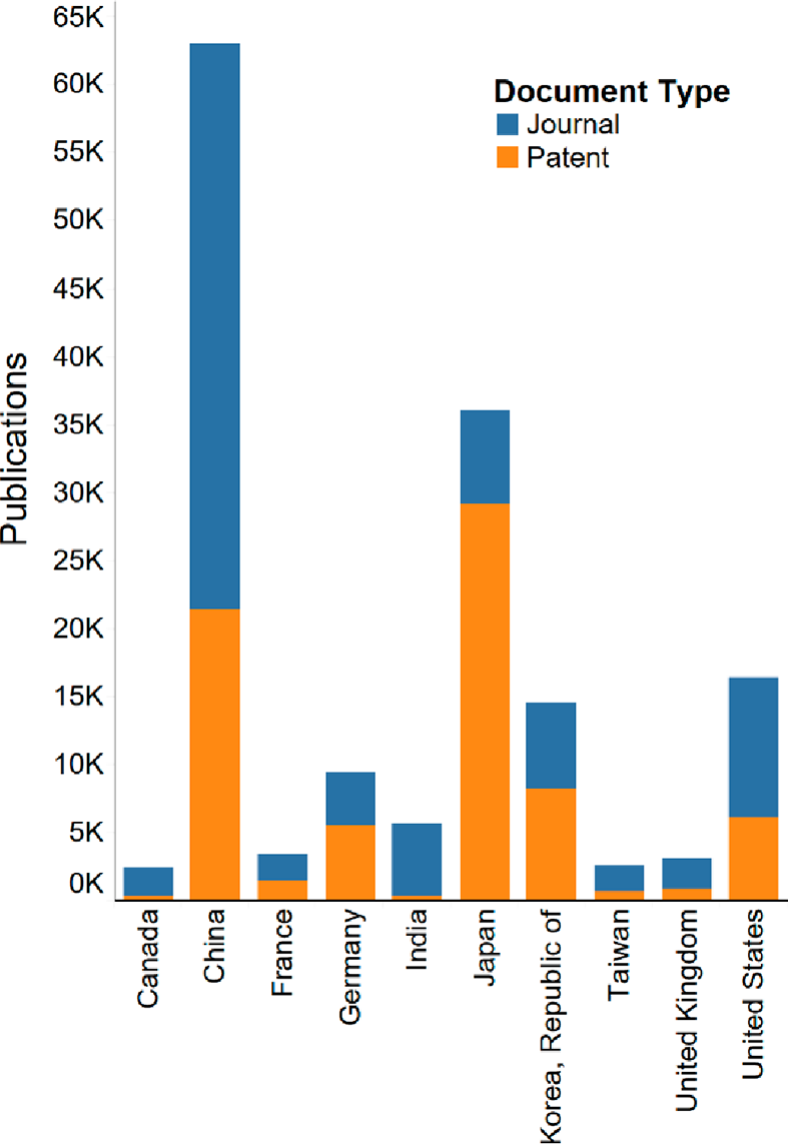
Journal and patent publications on the GHE by top organization countries/regions.

Our search of the GHE literature from 2011 to 2021 retrieved a total of 107 293 journal articles and 79 193 patents. Leading the way in the number of publications throughout the decade are China, Japan, the U.S., the Republic of Korea, and Germany (Figure 15).

**Figure 16:**
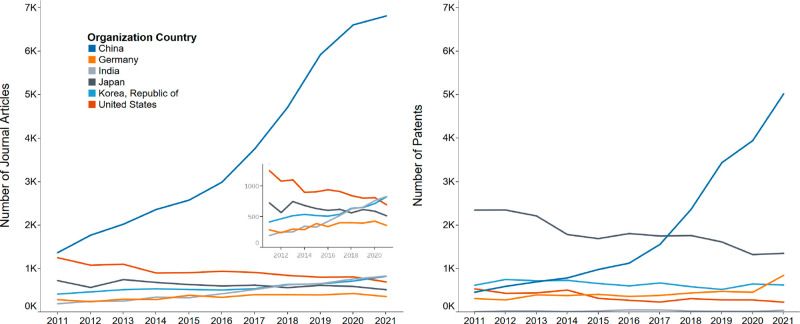
Journal articles and patents over time on the GHE for selected countries/regions.

China has the largest number of journal articles, and its publication numbers in general across the decade have an almost exponential growth and dwarf all other countries (Figure 16). Interest in green hydrogen has surged thanks to China’s drive to achieve carbon neutrality by 2060. Some recent real-world examples of China’s rising interest in GHE are the following: the city of Zhangjiakou inaugurated the world’s largest electrolyzer for green hydrogen production to provide fuel for hydrogen fuel cell vehicles during the 2022 Winter Olympics;^314^ Sinopec, China’s largest oil refiner, has started to build the world’s largest green hydrogen plant, to be entirely powered by solar energy;^315^ and a top Chinese solar manufacturer, Longi Green Energy Technology Co., has invested in the production of electrolyzers for green hydrogen production.^316^ Though China has yet to introduce a national hydrogen plan, the Chinese Hydrogen Alliance was launched in 2018 by China Energy Corporation and currently has 87 members including universities, research institutions, and large companies in the energy production and manufacturing sectors.^317^

Japan has had the most patents published throughout the decade (Figure 15). Though its patent publications are decreasing yearly (Figure 16), the country is investing significantly in hydrogen production and use. The government provided 370 billion yen ($3.4 billion) in funds to research, develop, support, and promote hydrogen with 70 billion yen allocated toward hydrogen production via water electrolysis.^318^ The interest in hydrogen is also promoted by the Japan Hydrogen Association, which includes 274 members between companies, municipalities, and universities.^319^ Japan promoted this technology thoroughly in the Tokyo 2020 Olympics, where the Olympic cauldron was lit with hydrogen, Toyota provided 500 Mirai fuel cell vehicles and 100 fuel cell buses for transportation, and hydrogen-based electricity was used in the Olympic village.^320^ Another example is the New Energy and Industrial Technology Development Organization’s (NEDO) green hydrogen plant, where 45 acres in Namei are now occupied by a solar farm where excess solar power is used for water electrolysis.^321^

The United States produced more journal articles than patents, but interest in the GHE decreased slightly throughout the decade (Figure 16). Still, we see that GHE publications will increase again because clean hydrogen is crucial to the U.S. Department of Energy’s strategy for achieving a 100% clean electrical grid by 2035 and net-zero carbon emissions by 2050, and the recent Bipartisan Infrastructure Law includes the following: $8 billion for Regional Clean Hydrogen Hubs to expand use of clean hydrogen, $1 billion for a Clean Hydrogen Electrolysis Program to reduce costs of green hydrogen production, and $500 million for Clean Hydrogen Manufacturing and Recycling Initiatives to support equipment manufacturing and strong domestic supply chains.^322^

There seems to be a larger academic interest in green hydrogen production as indicated by the large number of journal publications compared to patents (Figure 3 and Table 4). China comes in first place in number of publications, with its top patent assignee being the Dalian Institute of Chemical Physics, a council member of the Chinese Hydrogen Alliance. This aligns with the recent surge in interest of the solar and oil refining industries of China to build large electrolyzers and green hydrogen plants. The United States places second in number of publications, mostly in journals, followed by South Korea and Japan. Table 5 also demonstrates that no U.S. based company in the top assignees has patents, though Japan’s Toyota and JX Nippon Oil & Energy Corporation are tied for the second highest number of patents.

**Table 4:** Journal Articles and Patents on GHE by Top-Producing Countries/Regions from 2011 to 2021

	green hydrogen production		hydrogen storage		hydrogen fuel cells	
country/region	journal	patent	journal	patent	journal	patent
China	24 528	4 829	4 041	3 190	13 747	14 311
Japan	2 188	405	709	970	4 193	28 134
United States	3 785	356	842	391	6 093	5 492
Korea, Republic of	2 475	218	420	377	3 635	7 707
Germany	1 616	222	326	226	2 087	5 278
India	2 553	75	634	25	2 269	252
France	553	159	253	160	1 249	1 272
United Kingdom	786	42	222	44	1 279	806
Taiwan	762	28	123	60	1 080	621
Canada	582	32	179	22	1 336	338
Iran, Islamic Republic of	772	0	230	0	1 115	4
Italy	626	42	198	4	1 043	170
Russian Federation	494	54	202	32	950	185
Spain	661	20	123	6	817	82
Australia	701	22	206	20	389	66
Turkey	472	3	241	5	650	29
Switzerland	389	26	55	26	397	161

**Table 5:** Top Patent Assignees on GHE in Each Research Area from 2011 to 2021^a^

	number of patents		
assignee	green hydrogen production	hydrogen storage	hydrogen fuel cells
Toyota	37	205	6 768
Honda	22	28	2 893
Hyundai	7	41	1 964
Panasonic	21	47	1 651
Nissan	2	11	1 629
Bosch	24	14	1 171
Daimler	2	14	972
Kyocera Corp.	2	0	790
Dalian Institute of Chemical Physics, Chinese Academy of Sciences	61	37	626
Kia	3	19	670
NGK Insulators, Ltd.	0	2	566
JX Nippon Oil & Energy Corporation	37	27	483
Aisin Seiki Co., Ltd.	0	0	476
GM Global Technology Operations, Inc.	0	25	452
Toto Ltd.	6	0	462

a Multinational companies are combined under individual names.

The biggest producer of publications on hydrogen storage is China, followed by Japan, the U.S., and South Korea. The leading countries are shown to pay attention to both academic research and practical development. Toyota leads the way in patents, supporting its push in Japan for FCVs (Table 5). While China has had the most publications overall in the GHE space, Japan leads the way in fuel cell publications, followed by China, the U.S., South Korea, and Germany. Japan has the most patents, which aligns with 9 of the top 15 patent assignees being Japanese-based multinational companies (Table 4 and Table 5). China’s publications are split almost evenly between patents and journals. The U.S. comes in third with GM being its highest patent assignee, followed by South Korea with Hyundai taking the lead followed by Kia, and then Germany with Bosch and Daimler.

The automotive industry is leading the way in hydrogen fuel cell and storage patent publication, with the commercial use of hydrogen as a transportation fuel becoming a reality. Some examples of these patents in real life are Toyota’s Mirai, Honda’s Clarity, and Hyundai’s NEXO commercial FCVs available in the market, as well as FC trucks and buses like Hyundai’s XCient and Toyota’s Sora.^323−326^ Other manufacturing companies are joining in, with Panasonic just launching a 5 kW hydrogen fuel cell generator^327^ and its plan to build a large facility that uses pure hydrogen fuel cell generators (500 kW) as part of its in-house power for its fuel cell factory department at its Kusatsu site in Shiga Prefecture.^328^

## Conclusions and Outlook

In this Review, an analysis of GHE literature from 2011 to 2021 is discussed. A simplified discussion of the main types of electrolyzers and fuel cells with their advantages and disadvantages is provided. A description and comparison between physical hydrogen storage and materials-based hydrogen storage are given. A brief review of the recent technologies and materials used in the categories of green hydrogen production, hydrogen storage, and fuel cells is presented. Lists of the most commonly referenced substances in all three categories are provided with the mention of some of their features and literature usage. A more thorough discussion of hydrogen storage materials is presented.

Interest in green hydrogen production increased during the 2011–2021 decade as evidenced by its publication volumes. Publication volumes for hydrogen storage and hydrogen fuel cell research decreased early in the decade but increased later on, driven by increasing patent activity. These increasing patent volumes suggest that technologies for hydrogen storage and for fuel cells are more advanced than those for green hydrogen production, whose proportion of patents has yet to reach 20%. The number of catalyst materials studied for green hydrogen production has increased over the past decade, while the number of materials studied for use in hydrogen storage and fuel cell production has fallen, consistent with their relative levels of technical maturity. Nanotechnology concepts were shown to be popular in all three divisions of the GHE, particularly in hydrogen production. When combined with the increases in patent volume, it appears that hydrogen storage and fuel cells are closer to commercialization than green hydrogen production. Japan, China, and the automotive industry are the prime leaders in patent publications.

## Supporting Information Available

Data source bibliographic search strategies (PDF)
