# Supplementary material for: Materials Research Directions Toward a Green Hydrogen Economy: A Review
Source: ACS Omega. 2022 Sep 9;7(37):32908–35. doi: 10.1021/acsomega.2c03996 (PMC9494439; doi:10.1021/acsomega.2c03996)
Supplement: Supplementary file 1 — ao2c03996_si_001.pdf [file ao2c03996_si_001.pdf]

Supplement Information

Materials Research Directions Towards a Green Hydrogen Economy: A Review

Zachary J. Baum[‖], Leilani Lotti Diaz[‖], Tatyana Konovalova[‖] and Qiongqiong Angela Zhou*

[‖]These authors contribute equally to this paper.

Author Affiliation: CAS, a division of the American Chemical Society, Columbus, Ohio 43202, United States

*Corresponding author: Qiongqiong Angela Zhou qzhou@cas.org

Data source:

This work used data from CAS Content Collection, which covers publications in more than 50,000 scientific journals from around the world in a wide range of disciplines, 62 patent authorities, and 2 defensive publications (Research Disclosures and IP.com)[1]. There are more than 1000 global scientists specialized in various scientific domains curating, analyzing, and connecting data from published sources at CAS. The CAS Content Collection, as one of the largest collections of scientific databases in the world, has many unique features and annotations added during data curation.

Bibliographic search strategies:

The CAS Content Collection was searched to identify

(1) green hydrogen production-
(2) Hydrogen storage-
(3) Hydrogen fuel cells-

related publications from 2011-2021 based on various terms in their title, keywords, abstract text, and CAS expert-curated concepts. The search query required screening of each term to minimize false positives due to polysemy; a maximum of a 2% false positive rate was allowed for each OR-delimited phrase, as determined by random screenings of 300 documents performed by CAS experts. In addition, matches on particularly problematic phrases were excluded from consideration. Documents published in 2022 were not included in the trend analysis since the fiscal year is uncompleted. The resulting search strings are shown as below:

(1) Green hydrogen production:

((hydrogen evolution NOT batter?) OR water splitting OR ((H2 OR hydrogen OR alkaline OR proton exchange membrane OR solid oxide) and electrolyzer? NOT CO2 NOT carbon dioxide) OR (hydrogen production and ?catalys?) OR hydrogen evolution reaction catalysts/ct) NOT biosensor? NOT side(2A)reaction NOT wastewater NOT (a review OR review/dt OR overview) AND (journal/dt or patent/dt) AND 2011-2021/py

(2) Hydrogen storage:

((hydrogen storage AND (ammonia OR tank? OR apparatus OR adsorbent? OR container? OR hydride? OR alloy? OR borohydride? OR metal-organic framework? OR borane? OR MOF?)) OR hydrogen storage material?) NOT (a review OR review/dt OR overview) AND (journal/dt or patent/dt) AND 2011-2021/py

(3) Hydrogen fuel cells:

(fuel cell? Or fuel cells/ct) NOT (weapon or microbial or microbial alcohol fuel cell or methanol fuel cell or direct methanol or dmfc or hydrazine fuel cell or hydrogen peroxide fuel cell or methanol steam or gasification or syngas or natural gas or formic acid fuel cell or ammonium fuel cell or bio-gas or bio gas or bio energy or bio-energy or ethanol or glycerol or kerosene or jet fuel or gasoline or diesel) AND 2011-2021/py and (journal/dt or patent/dt) Not (a review or overview or review/dt)

1. *CAS Content*. https://www.cas.org/about/cas-content (accessed Feb 15, **2022**)
